# Flavonoids for Treating Viral Acute Respiratory Tract Infections: A Systematic Review and Meta-Analysis of 30 Randomized Controlled Trials

**DOI:** 10.3389/fpubh.2022.814669

**Published:** 2022-02-16

**Authors:** Jia Yao, Yuan Zhang, Xian-Zhe Wang, Jia Zhao, Zhao-Jun Yang, Yu-Ping Lin, Lu Sun, Qi-Yun Lu, Guan-Jie Fan

**Affiliations:** ^1^School of Second Clinical Medicine, Guangzhou University of Chinese Medicine, Guangzhou, China; ^2^Guangdong Provincial Hospital of Chinese Medicine, Guangzhou, China

**Keywords:** flavonoids, acute respiratory tract infection, COVID-19, complementary and alternative medicine, systematic review, meta-analysis

## Abstract

**Background:**

This meta-analysis aimed to investigate the efficacy and safety of flavonoids in treating viral acute respiratory tract infections (ARTIs).

**Methods:**

Randomized controlled trials (RCTs) were entered into meta-analyses performed separately for each indication. Efficacy analyses were based on changes in disease-specific symptom scores. Safety was analyzed based on the pooled data from all eligible trials, by comparing the incidence of adverse events between flavonoids and the control.

**Results:**

In this study, thirty RCTs (*n* = 5,166) were included. In common cold, results showed that the flavonoids group decreased total cold intensity score (CIS), the sum of sum of symptom intensity differences (SSID) of CIS, and duration of inability to work vs. the control group. In influenza, the flavonoids group improved the visual analog scores for symptoms. In COVID−19, the flavonoids group decreased the time taken for alleviation of symptoms, time taken for SARS-CoV−2 RT-PCR clearance, the RT-PCR positive subjects at day 7, time to achievement of the normal status of symptoms, patients needed oxygen, patients hospitalized and requiring mechanical ventilation, patients in ICU, days of hospitalization, and mortality vs. the control group. In acute non-streptococcal tonsillopharyngitis, the flavonoids group decreased the tonsillitis severity score (TSS) on day 7. In acute rhinosinusitis, the flavonoids group decreased the sinusitis severity score (SSS) on day 7, days off work, and duration of illness. In acute bronchitis, the flavonoids group decreased the bronchitis severity score (BSS) on day 7, days off work, and duration of illness. In bronchial pneumonia, the flavonoids group decreased the time to symptoms disappearance, the level of interleukin−6 (IL−6), interleukin−8 (IL−8), and tumor necrosis factor-α (TNF-α). In upper respiratory tract infections, the flavonoids group decreased total CIS on day 7 and increased the improvement rate of symptoms. Furthermore, the results of the incidence of adverse reactions did not differ between the flavonoids and the control group.

**Conclusion:**

Results from this systematic review and meta-analysis suggested that flavonoids were efficacious and safe in treating viral ARTIs including the common cold, influenza, COVID−19, acute non-streptococcal tonsillopharyngitis, acute rhinosinusitis, acute bronchitis, bronchial pneumonia, and upper respiratory tract infections. However, uncertainty remains because there were few RCTs per type of ARTI and many of the RCTs were small and of low quality with a substantial risk of bias. Given the limitations, we suggest that the conclusions need to be confirmed on a larger scale with more detailed instructions in future studies.

**Systematic Review Registration:** inplasy.com/inplasy-2021-8-0107/, identifier: INPLASY20218010

## Introduction

Acute respiratory tract infection (ARTI) is a common infectious disease and the most common reason for acute outpatient physician office visits. ARTI mainly includes viral infections, bacterial infections, and atypical pathogen infections, among which viral infections are the main cause of ARTIs. Viral ARTIs have become one of the key prevention and control diseases of infectious diseases in the world (1, 2). Common respiratory viruses include influenza virus, respiratory syncytial virus, adenovirus, parainfluenza virus, rhinovirus, human metapneumovirus, and human coronavirus. The symptoms of patients with viral ARTIs are different and not specific. The milder manifests as upper respiratory tract infection (URTI) symptoms such as cough, fever, and runny nose; the severer manifests as lower respiratory tract infection, which can induce wheezing diseases such as bronchiolitis, pneumonia, and asthma. Viral ARTIs can affect all systems of the whole body, and even cause death in severe cases.

The current treatment options for many viral ARTIs are typically symptom-related, but currently, there is no effective cure. The first-line treatment is rest, fluids, maintenance of hydration status, and prevention of viral/bacterial spread (3). Antibiotics are ineffective to treat viral infections, but analgesics and antipyretics can be prescribed and purchased over the counter to relieve symptoms such as pain and/or fever (3). Some viral ARTIs such as COVID−19 caused by severe acute respiratory syndrome coronavirus 2 (SARS-CoV−2) may use antivirals, anti-HIV protease inhibitors, anti-inflammatory agents, etc.; however, the effectiveness of most interventions of COVID−19 is uncertain because most of the randomized controlled trials (RCTs) so far have been small and have important limitations (4).

Besides the above “old” drugs, that clinicians are currently using against viral ARTIs, natural compounds isolated from the plant kingdom and belonging to the multiple and heterogeneous class of flavonoids may represent a useful option. Flavonoids are a major class of dietary polyphenols naturally occurring in herbs, plant-based foods, and beverages. Based on their chemical structure, flavonoids can be subclassified into six principal subclasses: flavonols (mainly including quercetin, kaempferol, myricetin, and isorhamnetin), flavones (apigenin and luteolin), flavanones (hesperetin and naringenin), flavan−3-ols (catechin, epicatechin, epigallocatechin, epicatechin−3-gallate, and epigallocatechin−3-gallate), anthocyanins (cyanidin, delphinidin, malvidin, pelargonidin, petunidin, and peonidin), and isoflavones (genistein and daidzein) (5–7). In the last two decades, much attention has been given to flavonoids and their proposed chemo-preventive bioactivities, especially their antiviral properties concerning viral infectious diseases (8–11). At present, flavonoids have been studied against a wide range of DNA and RNA viruses (12). Flavonoids are proposed to treat ARTIs because they have a range of physiologic effects in humans, including antiviral, anti-inflammatory, cytotoxic, antimicrobial, antioxidant, antiallergic, and antitumor activities (13–15). Flavonoids lack systemic toxicity, their ability to synergize with conventional drugs has been largely demonstrated, and finally, they are “pleiotropic” compounds, meaning that their functional groups can interact with different cellular targets and intercept multiple pathways. These features make flavonoids potential candidates to interfere with the viral life cycle (8). To date, there are some studies conducted on patients concerning the efficacy of flavonoids against respiratory tract infections. Preclinical studies have shown evidence of antiviral activity of quercetin-type flavonols with a significantly reduced mortality rate of infected animals and a reduction in the average viral load (16, 17). Furthermore, a meta-analysis in 2016 conducted by Somerville et al. included 14 studies and found that flavonoid supplementation decreased upper respiratory tract infections incidence by 33% compared with control in healthy adults, with no apparent adverse effects (18).

To date, there are some RCTs conducted on patients concerning the efficacy of flavonoids in treating viral ARTIs. However, the RCTs have shown inconsistent results and the evidence of the antiviral activity of flavonoids against viral ARTIs remains decentralized. Therefore, the purpose of this current systematic review and meta-analysis of the available evidence is to investigate the efficacy of flavonoids in treating viral ARTIs.

## Materials and Methods

The current systematic review and meta-analysis were reported following the Preferred Reporting Items for Systematic Reviews and Meta-Analyses (PRISMA) statement. INPLASY registration number was INPLASY202180107. Available from https://inplasy.com/inplasy-2021-8-0107/.

### Literature Search

Databases including Pubmed (https://pubmed.ncbi.nlm.nih.gov/), Embase (https://www.embase.com/), Web of Science (https://www.webofscience.com/), and the Cochrane Central Register of Controlled Trials (https://www.cochranelibrary.com/) were searched from inception time to September 16, 2021. The ClinicalTrials.gov registry was also searched for unpublished trials and the authors were contacted for additional information if necessary. We developed the search strategy with the assistance of an expert medical librarian, and the search terms were as follows: flavonoid, flavonol, flavone, flavanone, flavan−3-ol, anthocyanidin, isoflavone, viral acute respiratory tract infection, virus, respiratory tract infection, influenza, common cold, COVID−19, and randomized controlled trial. The search syntaxes used were detailed in the Supplementary Appendix. Relevant references from included studies were sought to retrieve additional eligible studies. No limits were set on language, publication year, and type of publication.

### Inclusion and Exclusion Criteria

The inclusion criteria were as follows: (1) RCTs published with any follow-up duration and sample size were included in this systematic review and meta-analysis; (2) Patients who suffered from virus-induced ARTIs; there was no restrictions for gender, age, or ethnicity; (3) The interventions for treating viral ARTIs in the experimental group could be any kinds of flavonoids; (4) Outcome measures: 1) In common cold, the primary outcome was the change of the total cold intensity score (CIS); the secondary outcomes included the change of the sum of symptom intensity differences (SSID) of CIS, clinical cure rate (CIS equals zero points or complete resolution of all but a maximum of one symptom) after treatment, major improved or completely recovered according to the integrative medicine outcome scale (IMOS) after treatment, the remission and improvement rates of symptoms on day five, mean duration of inability to work, the integrative medicine patient satisfaction scale (IMPSS), and the incidence of adverse reactions. 2) In influenza, the primary outcome was the change of visual analog scores (VAS) for symptoms; the secondary outcomes included time absent from work, duration of fever, duration of cough, complete cure on day 3, and the incidence of adverse reactions. 3) In COVID−19, the primary outcomes included time taken for alleviation of symptoms, time taken for SARS-CoV−2 RT-PCR clearance, patients in ICU, and mortality; the secondary outcomes included healed of symptoms on day 7, RT-PCR (positive subjects) on day 7, median clinical grading score (CGS) on day 6, not hospitalized with the resumption of normal activities on day 6, time to achievement of the normal status of symptoms, patients needed oxygen, hospitalized and requiring mechanical ventilation, days of hospitalization, changes of laboratory test index, and the incidence of adverse reactions. 4) In acute non-streptococcal tonsillopharyngitis, the primary outcome was the change of the tonsillitis severity score (TSS) on day 7; the secondary outcomes included major improved or completely recovered on the IMOS on day 4, the complete improvement rate of symptoms on day 4, the number of patients unable to work after treatment, IMPSS, and the incidence of adverse reactions. 5) In acute rhinosinusitis, the primary outcome was the change of the sinusitis severity score (SSS) on day 7; the secondary outcomes included major improved or completely recovered on the IMOS on day 7, the complete improvement rate of symptoms, radiographic cure on day 21, the number of patients unable to work on day 7, days-off work and duration of illness, IMPSS, and the incidence of adverse reactions. 6) In acute bronchitis, the primary outcome was the change of bronchitis severity score (BSS) on day 7; the secondary outcomes included major improved or completely recovered on the IMOS on day 7, the complete improvement rate of symptoms, days-off work, and duration of illness, the number of patients unable to work on day 7, IMPSS, and the incidence of adverse reactions. 7) In bronchial pneumonia, the primary outcome was time to symptom disappearance; the secondary outcomes included the changes of laboratory test index, the incident of complications, and the total incidence of adverse reactions. 8) In upper respiratory tract infections (no specific disease type was mentioned), the primary outcomes were the change of total CIS on day 7 and the improvement rate of symptoms; the secondary outcome was the incidence of adverse reactions.

The exclusion criteria were as follows: (1) outcome measures were not appropriate, relevant data could not be obtained from the original author; (2) non-randomized controlled trials, animal experiments, or review articles; and (3) repeated published literature.

### Data Extraction

For each eligible trial, pairs of reviewers (JY and YZ), following training and calibration exercises, extracted data independently by using a standardized, pilot-tested data extraction form. Reviewers collected information on trial characteristics (author, publication year, design, and sample size), patient characteristics (age and sex), experimental and control interventions, duration, and outcomes of interest. Reviewers resolved discrepancies by discussion and, when necessary, with adjudication by a third party (XW). When relevant details were insufficiently reported in studies, authors will be contacted by email, and the ClinicalTrials.gov register was searched for further information.

### Quality Assessment

For each eligible trial, reviewers, following training and calibration exercises, will use a revision of the Cochrane tool for assessing the risk of bias in randomized trials (RoB 2) (19) to rate trials as either at (1) low risk of bias, (2) some concerns—probably low risk of bias, (3) some concerns—probably high risk of bias, or (4) high risk of bias, across the following domains: randomization process, deviations from intended interventions, missing outcome data, measurement of the outcome, and selection of the reported result. Within each domain, the assessment comprised a series of signaling questions; a judgment about risk of bias for the domain, facilitated by an algorithm that maps responses to signaling questions to a proposed judgment; free text boxes to justify responses to the signaling questions and risk-of-bias judgments; and optional free text boxes to predict (and explain) the likely direction of bias. Furthermore, we assessed the risk of bias of included RCTs by using version 2 of the Cochrane risk-of-bias assessment tool for randomized trials (19). We rated trials at low risk of bias overall if the study was judged to be at low risk of bias for all domains for this result; rated trials at some concerns of bias overall if the study was judged to be at some concerns in at least one domain for this result, but not to be at high risk of bias for any domain; rated trials at high risk of bias overall if the study was judged to be at high risk of bias in at least one domain for this result or was judged to have some concerns for multiple domains in a way that substantially lowers confidence in the result. Reviewers resolved discrepancies by discussion and, when not possible, with adjudication by a third party.

### Statistical Analysis

Stata, version 16 (StataCorp LLC, College Station, TX, USA) was used for statistical analysis. Continuous data used the weighted mean difference (WMD) with 95% confidence intervals (CI) after the units are standardized. Dichotomous data used the relative risk (RR) with 95% CI. Missing data were dealt with according to the Cochrane Handbook for Systematic Reviews of Interventions. *P* < 0.05 was considered statistically significant. Heterogeneity was tested by χ^2^-based Cochran Q statistic (*P* < 0.10 indicated statistically significant heterogeneity) and *I*^2^ statistic. If *I*^2^ < 50%, a fixed-effects model was used to pool the estimations across studies. If *I*^2^ ≥ 50%, after excluding clinical heterogeneity between studies, the random-effects model was used. Quantitative data, where possible, were pooled for meta-analysis. Where pooling was not possible, the findings were presented in a narrative form. We tried to explain the source of heterogeneity by subgroup analysis or sensitivity analysis. Subgroup analyses were used to explore possible sources of heterogeneity, based on the following: (1) trial characteristics: sample size; (2) patient characteristics: the severity of COVID−19, age, whether the patient was hospitalized (inpatients or outpatients); and (3) types of intervention drugs. Sensitivity analysis was conducted by excluding studies one by one so that we can determine the source of heterogeneity. Publication bias was examined according to the funnel plot method. The Egger's test and Begg's test were conducted to quantitatively assess the publication bias, furthermore, the trim and fill method was used to correct the funnel asymmetry caused by publication bias.

## Results

### Search Results

**Figure 1:**
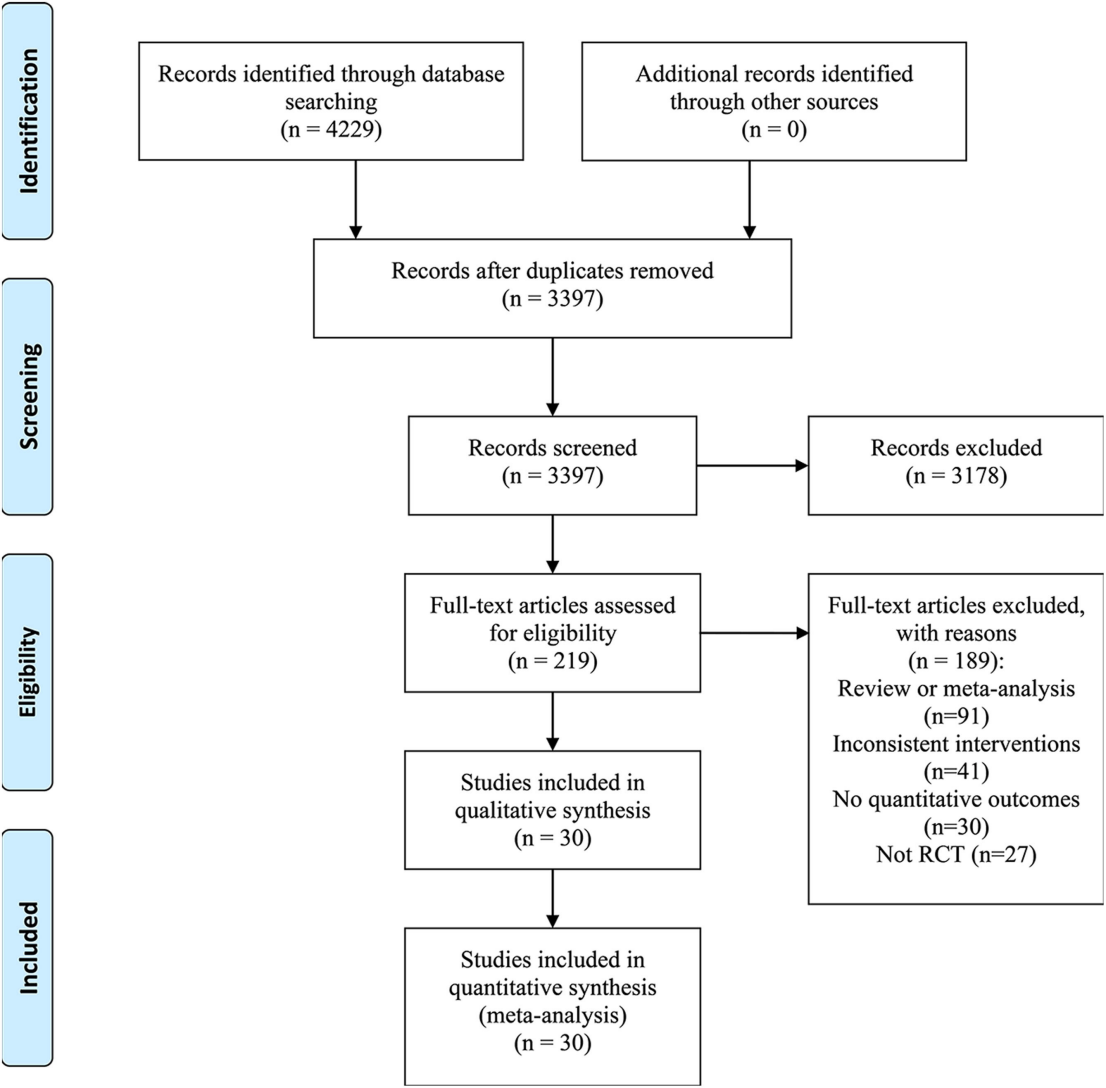
Flow diagram of study selection.

As displayed in Figure 1, we identified 4,229 citations in total with 832 duplicates. After preliminary screening of the titles and abstracts, 219 studies were selected for full-text review, and then 189 studies were excluded since 91 of them were reviews or meta-analyses, 27 of them were not RCTs, 30 studies did not provide quantitative outcomes, and the rest were those with undesirable interventions. Correspondence with the authors via e-mail was done to obtain the needed information for the study with no specific data on an outcome. Unfortunately, no reply from the authors was obtained until the time of this writing. Ultimately, 30 RCTs (20–49) were determined to be included in this systematic review and meta-analysis.

### Study Characteristics

Baseline characteristics of RCTs included in this systematic review and meta-analysis were shown in Table 1. Thirty studies involving 5,166 subjects (flavonoids group: 2636; control group: 2530) were included. Five eligible studies (20–24) investigated the efficacy of flavonoids in the common cold, 4 (25–28) in influenza, 5 (29–33) in COVID−19, 3 (34–36) in acute non-streptococcal tonsillopharyngitis, 1 (37) in acute rhinosinusitis, 7 (38–44) in acute bronchitis, 1 (45) in bronchial pneumonia, and 4 (46–49) in upper respiratory tract infections. In common cold, 5 RCTs with 503 patients were involved. The type of flavonoids included EPs 7630, verum, and troxerutin. Sample size ranged from 94 to 105 participants, duration varied from 4 to 10 days, mean age ranged from 27.6 to 40.8 years. In influenza, 4 RCTs with 571 patients were involved. The type of flavonoids included lozenge, echinaforce hotdrink, and sambucol. Sample size ranged from 27 to 420 participants, duration varied from 2 to 10 days, mean age ranged from 5 to 40.1 years. In COVID−19, 5 RCTs with 1,060 patients were involved. The type of flavonoids included honey plus nigella sativa, quercetin, and propolis. The sample size ranged from 42 to 429, duration varied from 7 days to 2 months. In acute non-streptococcal tonsillopharyngitis, 3 RCTs with 345 patients were involved. The type of flavonoid was EPs 7630. Sample size ranged from 78 to 143 participants, duration was 6 days, mean age ranged from 7.4 to 8 years. In acute rhinosinusitis, 1 RCT with 103 patients was involved. The type of flavonoid was EPs 7630, duration was 22 days. In acute bronchitis, 7 RCTs with 2033 patients were involved. The type of flavonoid was EPs 7630. Sample size ranged from 124 to 468 participants, duration was 7 days, mean age ranged from 8.7 to 41.8 years. In bronchial pneumonia, 1 RCTs with 180 patients were involved. The type of flavonoid was naringenin, duration was 5 days. In upper respiratory tract infections, 4 RCTs with 371 patients were involved. The type of flavonoids included propolis spray, CYSTUS052, and EPs7630. Sample size ranged from 28 to 160 participants, duration varied from 5 days to 8 weeks, mean age ranged from 2.5 to 46 years.

**Table 1:** Baseline characteristics of trials included in the analysis.

**Author, year**	**Group**	**Sample size**	**Intervention**	**Dosage**	**Route of Administr-ation**	**Durat-ion**	**Mean age (year)**	**Sex (M/F)**
**Common cold**								
Lizogub et al. (20)	T	52	EPS	3 × 30 drops daily	Drinking	10 d	34.5 ± 10.6	16/36
	C	51	Placebo				37.4 ± 10.5	16/35
Riley et al. (21)	T	52	EPs 7630	3 × 60 drops daily	Drinking	10 d	36.8 ± 9.9	14/38
	C	52	Placebo				33.8 ± 10.8	12/40
Riley et al. (22)	T	53	EPs 7630	3 × 40 mg daily	Taking capsules	10 d	35.0 ± 10.9	13/40
	C	52	Placebo				37.7 ± 10.5	11/41
Schütz et al. (23)	T	49	Verum	2 × 250 ml daily	Drinking	10 d	40.8 ± 12.8	/
	C	48	Placebo				40.8 ± 13.1	/
Turner et al. (24)	T	49	Troxerutin and Zn gluconate	Troxerutin 50 mg and Zn gluconate 25 mg daily	Taking capsules	4 d	28.3 ± 9.8	24/25
	C	45	Zn gluconate	Zn gluconate 10 mg daily			27.6 ± 9.2	20/25
**Influenza**								
Kong et al. (25)	T	32	Lozenge	4 × 175 mg proprietary elderberry extract daily	Taking capsules	2 d	40 (20–55)	17/15
	C	32	Placebo	Matched placebo			40.1 (27–59)	17/15
Rauš et al. (26)	T	203	Echinaforce Hotdrink	On the first 3 days, 5 × 5 ml, and 3 × 5 ml on the following 7 days; oseltamivir placebo capsules twice a day over 10 days	Drinking + Taking capsules	10 d	33.7 ± 13.9	109/94
	C	217	Oseltamivir	Echinaforce Hotdrink placebo and 5 days of oseltamivir verum capsules, followed by 5 days of oseltamivir placebo capsules, twice daily			36.7 ± 13.1	101/116
Zakay-Rones et al. (27)	T	15	Sambucol	Children received two, and adults four tablespoons daily	Drinking	3 d	5–50	9/6
	C	12	Placebo				7–56	9/3
Zakay-Rones et al. (28)	T	30	Sambucol	4 × 15 ml daily	Drinking	5 d	30.6 ± 2.9	18/12
	C	30	Placebo				29.4 ± 2.8	15/15
**COVID−19**								
Ashraf et al. (29)	T	157	Honey + Nigella sativa	Honey (1 gm/Kg/day) and Nigella sativa seeds (80 mg/Kg/day)	Drinking + Taking capsules	13 d	≤40 (48.4%); 40–59 (30.57%); 60–79 (16.56%); ≥80 (4.45%)	90/67
	C	156	Placebo	Matched placebo	Taking capsules		≤40 (51.82%); 40–59 (28.85%); 60–79 (16.67%); ≥80 (3.2%)	88/68
Onal et al. (30)	T	49	QCB (Quercetin, vitamin C, bromelain) + Standard treatment	1,000 mg quercetin, 1,000 mg vitamin C and 100 mg bromelain daily in 2 divided doses	Taking capsules	2 m	18–30 (0%); 30–40 (2%); 40–50 (18.4%); 50–60 (32.7%); 60–70 (26.5%); 70–80 (16.3%); 80–90 (4.1%); 90–100 (0%)	32/17
	C	380	Standard treatment	Hydroxychloroquine, 400 mg daily for 5 days, and favipiravir, 2 × 600 mg for 4 days following a 2 × 1600 mg loading dose on day one			18–30 (5.3%); 30–40 (10%); 40–50 (20.5%); 50–60 (24.2%); 60–70 (20.5%); 70–80 (10.3%); 80–90 (7.6%); 90–100 (1.6%)	210/170
Di Pierro et al. (31)	T	21	Quercetin Phytosome + Standard treatment	The first 7 days 3 tablets daily, the following 7 days 2 tablets daily	Taking capsules	2 w	42.5 ± 3.3	10/11
	C	21	Standard treatment	To be performed at home, constituted by analgesics/anti-fevers and antibiotics			56.2 ± 3.3	10/11
Di Pierro et al. (32)	T	76	Quercetin Phytosome + Standard treatment	1,000 mg daily	Taking capsules	1 m	18–20 (5.3%); 21–30 (21.1%); 31–40 (17.1%); 41–50 (23.7%); 51–60 (22.4%); 61–70 (9.2%); 71–80 (1.2%)	42/34
	C	76	Standard treatment	Constituted by analgesics/anti-fevers, oral steroids, and anti-biotics			18–20 (6.6%); 21–30 (18.4%); 31–40 (18.4%); 41–50 (22.4%); 51–60 (23.7%); 61–70 (7.9%); 71–80 (2.6%)	46/30
Silveira et al. (33)	T	40	Propolis 400 mg/d	Propolis 400 mg daily plus standard care	Taking capsules	7 d	49.5 ± 12.8	28/12
	T	42	Propolis 800 mg/d	Propolis 800 mg daily plus standard care			48.9 ± 11.2	30/12
	C	42	Standard care	All necessary interventions, as determined by the attending physician			51.6 ± 14.3	28/14
**Acute Non-streptococcal tonsillopharyngitis**								
Berezhnoi et al. (34)	T	60	EPs 7630	3 × 20 drops daily	Drinking	6 d	7.6 ± 1.1	29/31
	C	64	Placebo				7.4 ± 1.2	28/36
Berezhnoy et al. (35)	T	73	EPs 7630	3 × 20 drops daily	Drinking	6 d	7.6 ± 1.3	40/33
	C	70	Placebo				7.5 ± 1.1	30/40
Timen et al. (36)	T	40	EPs 7630	20 drops hourly on the first 2 days while awake and thereafter 3 × 20 drops daily for a further 4 days	Drinking	6 d	8.0 ± 1.0	/
	C	38	Placebo					
**Acute rhinosinusitis**								
Bachert et al. (37)	T	51	EPs 7630	3 × 60 drops daily	Drinking	22 d	34.3 ± 10.3	19/32
	C	52	Placebo				35.6 ± 12.8	17/35
**Acute bronchitis**								
Chuchalin et al. (38)	T	64	EPs 7630	3 × 30 drops daily	Drinking	7 d	36.2 ± 13.0	15/49
	C	60	Placebo				35.9 ± 13.2	22/38
Kamin et al. (39)	T	103	EPs 7630	1–6 years: 3 × 10 drops daily; > 6–12 years: 3 × 20 drops daily; > 12–18 years: 3 × 30 drops daily	Drinking	7 d	9.4 ± 5.0	50/53
	C	97	Placebo				9.5 ± 5.1	45/52
Kamin et al. (40)	T	100	EPs 7630 30 mg/d	3 × 10 mg daily	Taking capsules	7 d	12.5 ± 3.5	53/47
	T	99	EPs 7630 60 mg/d	3 × 20 mg daily			12.9 ± 3.7	51/48
	T	99	EPs 7630 90 mg/d	3 × 30 mg daily			12.6 ± 3.7	52/47
	C	101	Placebo	Matched placebo			12.7 ± 3.7	51/50
Kamin et al. (41)	T	111	EPs 7630	3 × 10 drops (1–6 years old), 3 × 20 drops (6–12 years old) or 3 × 30 drops (12–18 years old) daily	Drinking	7 d	8.7 ± 4.8	54/57
	C	109	Placebo				9.2 ± 5.2	55/54
Matthys et al. (42)	T	233	EPs 7630	3 × 30 drops daily	Drinking	7 d	41.1 ± 14.1	94/136
	C	235	Placebo				39.9 ± 14.2	75/160
Matthys et al. (43)	T	108	EPs7630	3 × 30 drops daily	Drinking	7 d	37.4 ± 12.4	30/78
	C	109	Placebo					31/86
Matthys et al. (44)	T	102	EPs 7630 30 mg/d	3 × 10 mg daily	Taking capsules	7 d	40.3 ± 12.2	32/70
	T	101	EPs 7630 60 mg/d	3 × 20 mg daily			41.8 ± 13.2	24/77
	T	100	EPs 7630 90 mg/d	3 × 30 mg daily			38.8 ± 13.7	28/72
	C	102	Placebo	3 times daily			38.5 ± 12.6	39/63
**Bronchial pneumonia**								
Yao et al. (45)	T	90	Naringenin	5 mg/kg daily	Taking capsules	5 d	2.7 ± 1.2	42/48
	C	90	Azithromycin	10 mg/kg daily			2.6 ± 1.0	44/46
**Upper respiratory tract infections**								
Esposito et al. (46)	T	58	Propolis spray	2–4 sprays three times daily for 5 days	Oral spray	8 w	44.0 ± 14.0	29/29
	C	64	Placebo				44.0 ± 5.0	25/39
Kalus et al. (47)	T	80	CYSTUS052	6 × 2 tablets daily	Taking capsules	7 d	46 (10–77)	/
	C	80	Placebo				43 (7–81)	/
Patiroglu et al. (48)	T	14	EPs 7630	3 × 10 drops daily	Drinking	7 d	2.5 (1–5)	11/3
	C	14	Placebo					9/5
Tahan et al. (49)	T	30	EPs7630+ supportive treatment	3 × 10 drops (1–5 years old), 3 × 20 drops (6–12 years old) or 3 × 30 drops (>12 years old) daily	Drinking or taking capsules	5 d	5 (1–12)	15/15
	C	31	Supportive treatment	Paracetamol when needed			7 (1–14)	19/12

*M/F, male/female; T, treatment group; C, control group; d, day; m, month; w, week*.

### Quality Assessment

**Figure 2:**
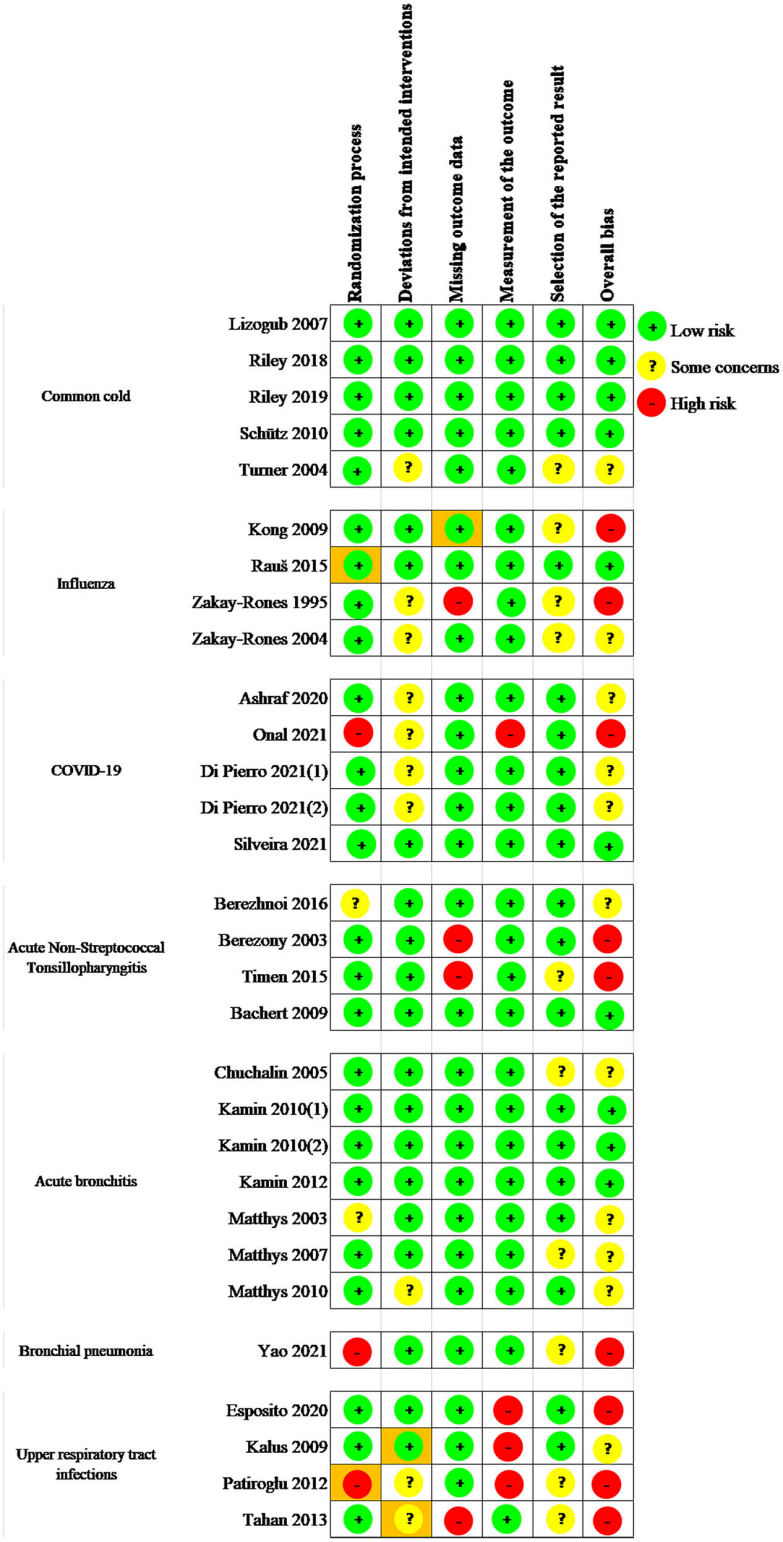
Risk of bias assessment in the included studies.

The risk of bias data for the included 30 RCTs was presented in Figure 2. The randomization process was rated at high risk in 3 (10%) RCTs (30, 45, 48) because concealment of allocation sequence was not presented or open-label design. Besides, 2 (6.7%) RCTs (34, 42) were rated at some concerns for the lack of details of the concealment and the difference in baseline data between groups; the remaining 25 (83.3%) RCTs were rated at low risk. Regarding the basis of deviations from intended interventions, 10 (33.3%) studies (24, 27–32, 44, 48, 49) did not elaborate on the analysis method used to estimate the effect of assignment for intervention clearly; among them, 5 (16.7%) RCTs were open-labeled trials, so participants, careers, and people delivering the interventions were aware of the assigned intervention during the trial. Thus, the 10 (33.3%) RCTs were categorized as some concerns. The remains were categorized as low risk. Regarding the basis of missing outcome data, 4 (13.3%) studies (27, 35, 36, 49) were considered as high risk for more than 5% of missing outcome data which could have made a negative impact on the estimated effect of the intervention. Regarding the bias of measurement of the outcome was rated at high risk in 4 (13.3%) RCTs (30, 46–48) for that the outcome assessors were aware of the interventions. Regarding the bias of selection of the reported result, 10 (33.3%) RCTs (24, 25, 27, 28, 36, 38, 43, 45, 48, 49) were rated at some concerns for no protocols or registrations. The remaining 20 (66.7%) RCTs were rated at low risk.

### Pooled Results

#### Common Cold

Five eligible RCTs (20–24) investigated the efficacy and safety of flavonoids in the common cold.

(1) Result of the primary outcome: change of the total CIS

**Figure 3:**
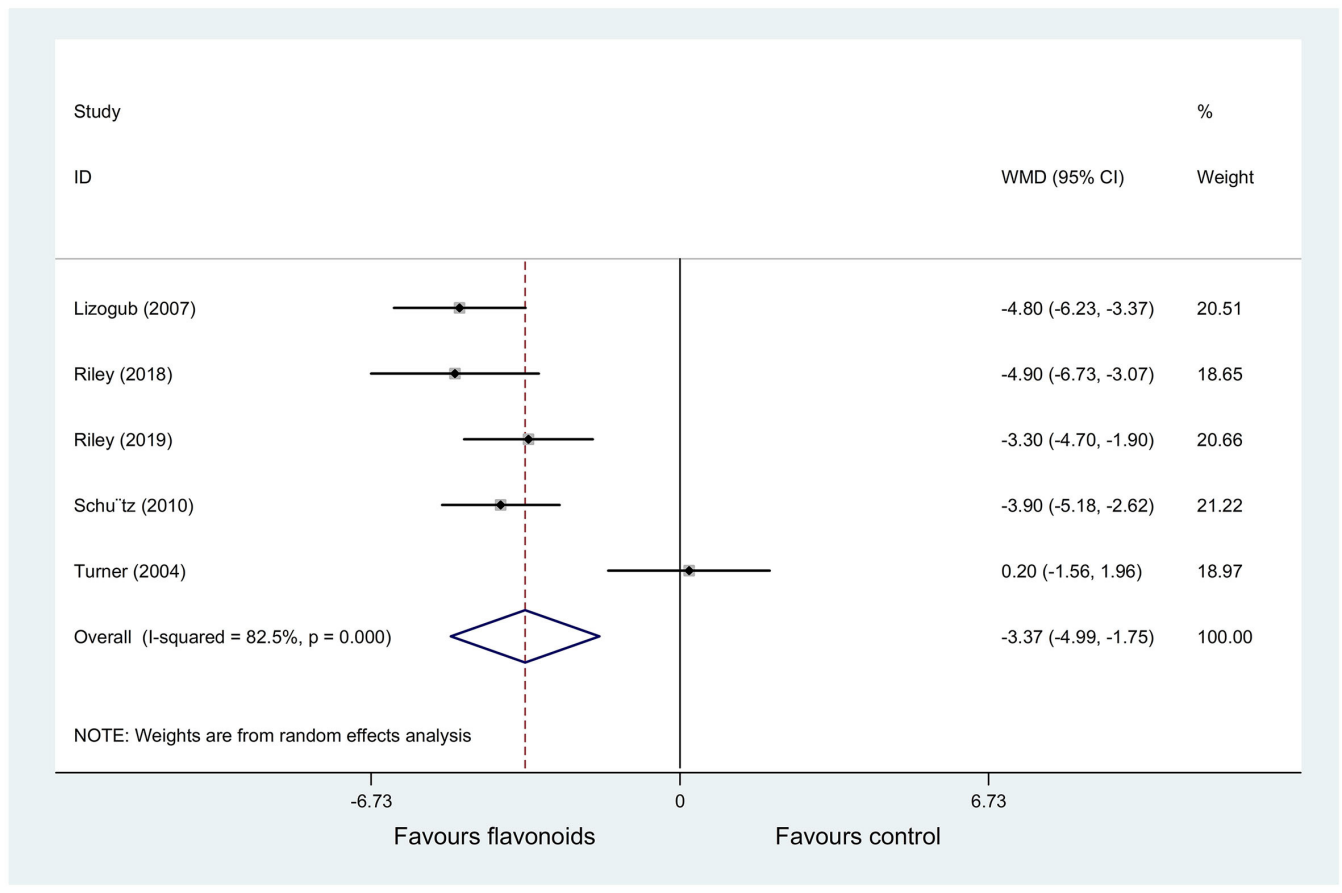
Meta-analysis of change of the total cold intensity score (CIS) in patients with the common cold.

Five RCTs (20–24) (*n* = 503) reported change of the total CIS as an outcome, and significant heterogeneity was observed (*P* < 0.001; *I*^2^ = 82.5%). Pooled results with a random-effects model showed that the flavonoids group significantly decreased the total CIS vs. the control group (WMD = −3.37, 95% CI: −4.99 to −1.75, *P* < 0.001) (Figure 3).

(2) Results of the secondary outcomes

As for secondary outcomes (Table 2), pooled results of 4 RCTs (20–23) (*n* = 409) with a random-effects model showed that the flavonoids group significantly decreased the sum of SSID of CIS vs. the control group (*P* = 0.026, *I*^2^ = 67.6%; WMD = −5.34, 95% CI: −7.26 to −3.42, *P* < 0.001). Furthermore, the flavonoids group exhibited a significantly improved clinical cure rate after treatment vs. the control group (*P* = 0.392, *I*^2^ = 0%; RR = 3.26, 95% CI: 2.49 to 4.28, *P* < 0.001). Pooled results of 3 RCTs (20–22) with a random-effects model showed that the flavonoids group significantly increased the major improved or completely recovered patients according to the IMOS after treatment vs. the control group (*P* = 0.1, *I*^2^ = 56.6%; RR = 5.15, 95% CI: 2.46 to 10.77, *P* < 0.01). Pooled results of 2 RCTs (20, 21) showed that the flavonoids group significantly increased the remission and improvement rates of symptoms including chills, limb pain, weakness, exhaustion, and fatigue on day 5 vs. the control group (RR = 1.13, 95% CI: 1.01 to 1.27, *P* = 0.031; RR = 1.29, 95% CI: 1.09 to 1.53, *P* = 0.003; RR = 2.03, 95% CI: 1.58 to 2.6, *P* < 0.001; RR = 1.82, 95% CI: 1.47 to 2.26, *P* < 0.001; RR = 1.49, 95% CI: 1.25 to 1.78, *P* < 0.001; respectively). Pooled results of 2 RCTs (20, 21) with a fixed-effects model showed that the flavonoids group significantly decreased the mean duration of inability to work compared with the control group (*P* = 0.259, *I*^2^ = 21.4%; WMD = −1.62, 95% CI: −2.14 to −1.1, *P* < 0.001). Results of 4 RCTs (20–23) with a fixed-effects model showed that the flavonoids group significantly increased IMPSS compared with the control group (*P* = 0.565, *I*^2^ = 0%; RR = 2.25, 95% CI: 1.86 to 2.73, *P* < 0.001). Pooled results of 5 RCTs (20–25) with 503 patients using a fixed-effects model showed that the incidence of adverse reactions did not differ between the flavonoids group and the control group (*P* = 0.339, *I*^2^ = 11.7%; RR = 1.58, 95% CI:0.92 to 2.71, *P* = 0.097).

**Table 2:** Results of the secondary outcomes in common cold.

**Outcomes**		**No. of trial**	**WMD/RR**	**95% CI**	***P***	***I^**2**^*; *P***	**Effect model**
Change of the sum of SSID of CIS		4	−5.34	−7.26 to −3.42	<0.001	67.6%; 0.026	RE
Clinical cure rate after treatment		4	3.26	2.49 to 4.28	<0.001	0.0%; 0.392	FE
Major improved or completely recovered according to the IMOS after treatment		3	5.15	2.46 to 10.77	<0.001	56.6%; 0.100	RE
The remission and improvement rates of symptoms on day five	Chills	1	1.13	1.01 to 1.27	0.031	/	FE
	Limb pain	1	1.29	1.09 to 1.53	0.003	/	FE
	Weakness	2	2.03	1.58 to 2.60	<0.001	0.0%; 0.493	FE
	Exhaustion	2	1.82	1.47 to 2.26	<0.001	0.0%; 0.524	FE
	Fatigue	2	1.49	1.25 to 1.78	<0.001	0.0%; 0.961	FE
Mean duration of inability to work		2	−1.62	−2.14 to −1.10	<0.001	21.4%; 0.259	FE
IMPSS		4	2.25	1.86 to 2.73	<0.001	0.0%; 0.565	FE
Incidence of adverse reactions		5	1.58	0.92 to 2.71	0.097	11.7%; 0.339	FE

*SSID, sum of symptom intensity differences; CIS, cold intensity score; IMOS, integrative medicine outcome scale; IMPSS, integrative medicine patient satisfaction scale; FE, fixed-effects model; RE, random-effects model*.

#### Influenza

Four RCTs (25–28) investigated the efficacy and safety of flavonoids in influenza.

(1) Result of the primary outcome: the change of VAS for symptoms

**Figure 4:**
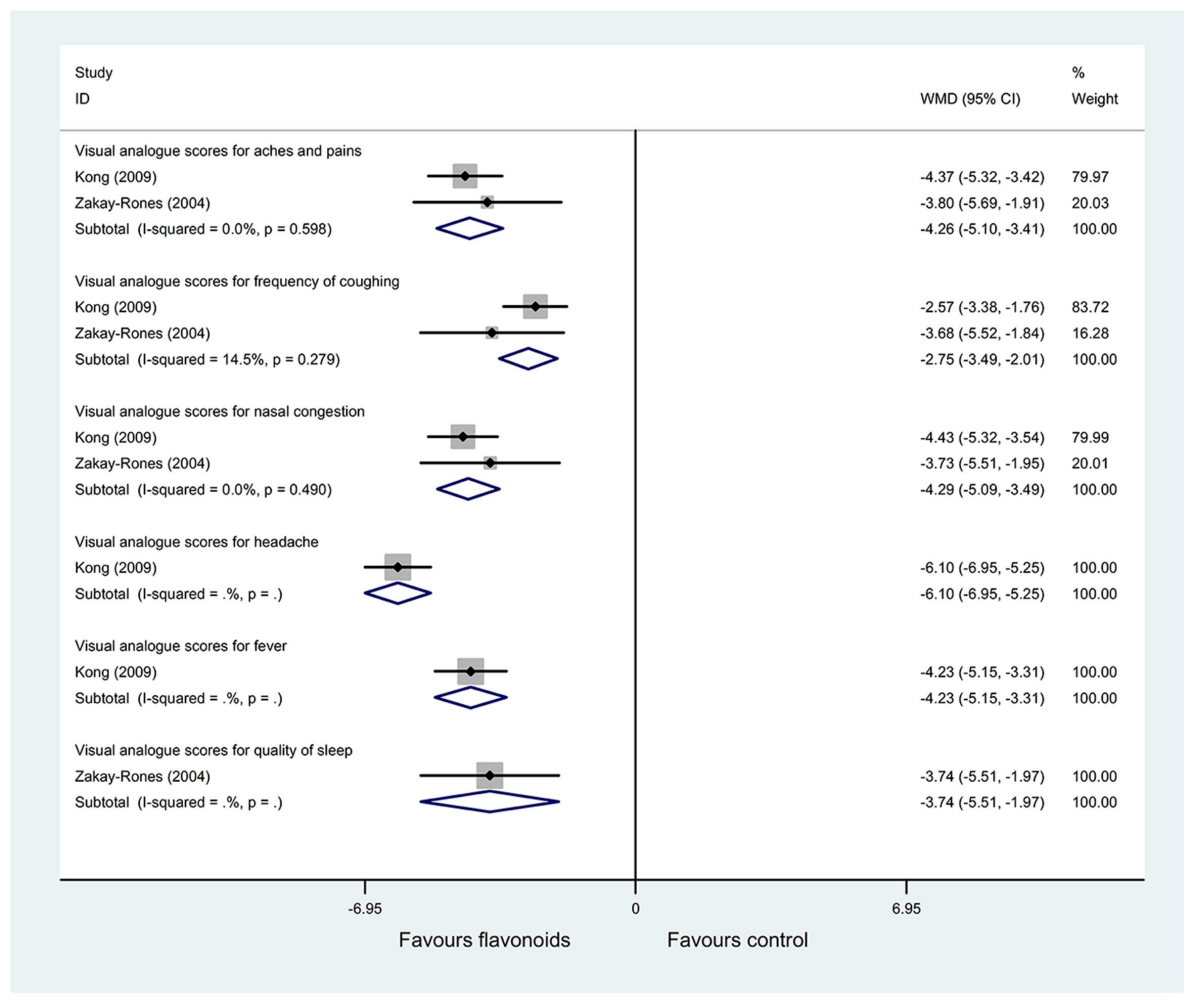
Meta-analysis of change of visual analog scores (VAS) for symptoms in patients with influenza.

Two RCTs (25, 28) with (*n* = 124) reported the change of VAS for symptoms as an outcome. Pooled results with a fixed-effects model showed that the flavonoids group significantly improved the VAS for aches and pains, frequency of coughing, nasal congestion, headache, fever, quality of sleep, and mucus discharge in the respiratory tract, and compared with the control group (WMD = −4.26, 95% CI: −5.10 to 3.41, *P* < 0.001; WMD = −2.75, 95% CI: −3.49 to −2.01, *P* < 0.001; WMD = −4.29, 95% CI: −5.09 to −3.49, *P* < 0.001; WMD = −6.1, 95% CI: −6.95 to −5.25, *P* < 0.001; WMD = −4.23, 95% CI: −5.15 to −3.31, *P* < 0.001; WMD = −3.74, 95% CI: −5.51 to −1.97, *P* < 0.001; WMD = −2.75, 95% CI: −4.06 to −1.43, *P* < 0.001; respectively) (Figure 4).

(2) Results of the secondary outcomes

As for secondary outcomes (Table 3), pooled results of 2 RCTs (26, 27) (*n* = 447) with a fixed-effects model showed that the flavonoids group significantly increased complete cure on day 3 compared with placebo (RR = 2.6, 95% CI: 1.14 to 5.93, *P* = 0.023); however, the complete cure on day 3 did not differ between the flavonoids and oseltamivir group (RR = 1.02, 95% CI: 0.71 to 1.47, *P* = 0.9). Furthermore, the outcomes of time absent from work, the duration of fever or cough, and the incidence of adverse reactions did not differ between the flavonoids group and the control group.

**Table 3:** Results of the secondary outcomes in influenza.

**Outcomes**		**No. of trial**	**WMD/RR**	**95% CI**	***P***	***I^**2**^*; *P***	**Effect model**
Complete cure on day 3	Flavonoids vs. placebo	1	2.60	1.14 to 5.93	0.023	/	FE
	Flavonoids vs. oseltamivir	1	1.02	0.71 to 1.47	0.900	/	FE
Time absent from work		1	−0.10	−0.38 to 0.18	0.483	/	FE
Duration of fever		2	−2.48	−5.45 to 0.49	0.101	94.5%; <0.001	RE
Duration of cough		1	0.20	−0.11 to 0.51	0.203	/	FE
Incidence of adverse reactions		3	0.382	0.14 to 1.04	0.06	/	FE

*FE, fixed-effects model; RE, random-effects model*.

#### COVID−19

Five RCTs (29–33) investigated the efficacy and safety of flavonoids in COVID−19. Pooled results were shown in Table 4, and subgroup analysis results were shown in Supplementary Tables 1, 2.

**Table 4:** Results of outcomes in COVID−19.

**Outcomes**		**No. of trial**	**WMD/ RR**	**95% CI**	***P***	***I^**2**^*; *P***	**Effect model**
**Primary outcomes**							
Time taken for alleviation of symptoms		1	−4.92	−7.46 to −2.37	<0.001	93.3%; <0.0001	RE
Time taken for SARS-CoV−2 RT-PCR clearance		1	−4.11	−4.54 to −3.68	<0.001	44.4%; 0.180	FE
Patients in ICU		3	0.289	0.14 to 0.61	0.001	2.5%; 0.359	FE
Mortality		4	0.30	0.12 to 0.78	0.013	0.0%; 0.679	FE
**Secondary outcomes**							
Healed of symptoms on day 7		1	3.00	1.15 to 7.81	0.024	/	FE
RT-PCR (positive subjects) on day 7		1	0.26	0.12 to 0.57	0.001	/	FE
Median CGS on day 6		1	−1.59	−2.77 to −4.00	0.009	95.3%; <0.001	RE
Not hospitalized with resumption of normal activities at day 6		1	6.66	3.82 to 11.60	<0.001	0.0%; 0.38	FE
Time to achievement of normal status of symptoms	fever	1	−4.00	−4.64 to −3.36	<0.001	0.0%; 1.000	FE
	cough	1	−2.63	−3.49 to −1.76	<0.001	16.8%; 0.273	FE
	myalgia	1	−2.72	−4.61 to −0.84	0.005	65.7%; 0.088	RE
Patients needed oxygen		2	0.19	0.09 to 0.41	<0.001	0.0%; 0.409	FE
Hospitalized and requiring mechanical ventilation		2	0.31	0.12 to 0.77	0.012	0.0%; 0.986	FE
Days of hospitalization		2	−5.09	−5.76 to −4.41	<0.001	21.6%; 0.259	FE
Changes of laboratory test index	CRP	3	−0.68	−0.86 to −0.48	<0.001	45.3%; 0.140	FE
	LDH	2	−50.83	−84.07 to −17.59	0.003	47.2%; 0.169	FE
	D-dimer	2	0.61	0.34 to 0.89	<0.001	0.0%; 0.637	FE
	ferritin	2	−89.48	−204.94 to 25.99	0.129	0.0%; 0.968	FE

*ICU, intensive care unit; CGS, clinical grading score; CRP, C-reactive protein; LDH, lactate dehydrogenase; FE, fixed-effects model; RE, random-effects model*.

(1) Results of the primary outcomes
1) Time is taken for alleviation of symptoms

Pooled result of 1 RCT (29) (*n* = 313) with a random-effects model showed that the flavonoids group significantly decreased the time taken for alleviation of symptoms compared with the control group (*P* < 0.0001, *I*^2^ = 93.3%; WMD = −4.92, 95% CI: −7.46 to −2.37, *P* < 0.001). Subgroup analysis was performed based on the severity of COVID−19 (moderate or severe cases), the results of each subgroup analysis showed that the flavonoids group significantly decreased the time taken for alleviation of symptoms vs. the control group (WMD = −3.7, 95% CI: −3.9 to −3.5, *P* < 0.001; WMD = −6.3, 95% CI: −7.6 to −5, *P* < 0.001; respectively) (Supplementary Table 1).

2) Time is taken for SARS-CoV−2 RT-PCR clearance

Pooled result of 1 RCT (29) (*n* = 313) with a fixed-effects model showed that the flavonoids group significantly decreased the time taken for SARS-CoV−2 RT-PCR clearance vs. the control group (*P* = 0.18, *I*^2^ = 44.4%; WMD = −4.11, 95% CI: −4.54 to −3.68, *P* < 0.001).

3) Patients in ICU

Pooled results of 3 RCTs (31–33) (*n* = 318) showed that the flavonoids group significantly decreased patients in ICU vs. the control group (*P* = 0.359, *I*^2^ = 2.5%; RR = 0.289, 95% CI:0.14 to 0.61, *P* = 0.001). Subgroup analysis was performed based on whether the patient was hospitalized (inpatients or outpatients), the results of each subgroup analysis showed that the flavonoids group significantly decreased patients in ICU vs. the control group (RR = 0.42, 95% CI:0.19 to 0.93, *P* = 0.033; RR = 0.1, 95% CI:0.01 to 0.77, *P* = 0.027; respectively) (Supplementary Table 2).

4) Mortality

Pooled results of 4 RCTs (29–32) (*n* = 934) showed that the flavonoids group significantly decreased mortality vs. the control group (*P* = 0.679, *I*^2^ = 0%; RR = 0.3, 95% CI:0.12 to 0.78, *P* = 0.013). Results of subgroup analysis showed that the flavonoids group significantly decreased mortality vs. the control group in inpatients subgroup (RR = 0.34, 95% CI:0.12 to 0.99, *P* = 0.048); however, in outpatients' subgroup, mortality did not differ between flavonoids group and the control (RR = 0.2, 95% CI:0.02 to 1.68, *P* = 0.138) (Supplementary Table 2).

(2) Results of the secondary outcomes
1) Healed of symptoms on day 7

Results of 1 RCT (31) (*n* = 42) with a fixed-effects model showed that the flavonoids group significantly increased the healed of symptoms on day 7 vs. the control group (RR = 3, 95% CI: 1.15 to 7.81, *P* = 0.024).

2) RT-PCR (positive subjects) on day 7

Results of 1 RCT (31) (*n* = 42) with a fixed-effects model showed that the flavonoids group significantly decreased the RT-PCR (positive subjects) on day 7 compared with the control group (RR = 0.26, 95% CI:0.12 to 0.57, *P* = 0.001).

3) CGS on day 6

Results of 1 RCT (29) (*n* = 313) with a random-effects model showed that the flavonoids group significantly decreased the median CGS at day 6 compared with the control group (*P* < 0.001, *I*^2^ = 95.3%; WMD = −1.59, 95% CI: −2.77 to −4, *P* = 0.009). Subgroup analysis was performed based on the severity of COVID−19 (moderate or severe cases), the results of each subgroup analysis showed that the flavonoids group significantly decreased the median CGS on day 6 vs. the control group (WMD = −1, 95% CI: −1.2 to −0.8, *P* < 0.001; WMD = −2.21, 95% CI: −2.68 to −1.74, *P* < 0.001; respectively) (Supplementary Table 1).

4) Not hospitalized with the resumption of normal activities on day 6

Result of 1 RCT (29) (*n* = 313) with a fixed-effects model showed that the flavonoids group significantly increased not hospitalized with the resumption of normal activities on day 6 compared with the control group (*P* < 0.38, *I*^2^ = 0%; WMD = 6.66, 95% CI: 3.82 to 11.6, *P* < 0.001).

5) Time to achievement of normal status of symptoms (fever, cough, and myalgia)

Results of 1 RCT (29) (*n* = 313) showed that the flavonoids group significantly decreased time to achievement of normal status of fever, cough, and myalgia vs. the control group (WMD = −4, 95% CI: −4.64 to −3.36, *P* < 0.001; WMD = −2.63, 95% CI: −3.49 to −1.76, *P* < 0.001; WMD = −2.72, 95% CI: −4.61 to −0.84, *P* = 0.005; respectively). Furthermore, significant heterogeneity was observed in the outcome of time to achievement of normal status of myalgia (*I*^2^ = 65.7%), subgroup analysis was performed based on the severity of COVID−19 (moderate or severe cases), the results of each subgroup analysis showed that the flavonoids group significantly decreased time to achievement of normal status of myalgia vs. the control group (WMD = −2, 95% CI: −2.71 to −1.29, *P* < 0.001; WMD = −4, 95% CI: −6.19 to −1.82, *P* < 0.001; respectively) (Supplementary Table 1).

6) Patients needed oxygen

Pooled results of 2 RCTs (29, 32) (*n* = 465) showed that the flavonoids group significantly decreased patients needed oxygen compared with the control group (*P* = 0.409, *I*^2^ = 0%; RR = 0.19, 95% CI:0.09 to 0.41, *P* < 0.001). Results of each subgroup analysis (inpatients or outpatients) showed that the flavonoids group significantly decreased patients needed oxygen vs. the control group (RR = 0.27, 95% CI:0.12 to 0.63, *P* = 0.002; RR = 0.07, 95% CI:0.01 to 0.49, *P* = 0.008; respectively) (Supplementary Table 2).

7) Patients hospitalized and requiring mechanical ventilation

Pooled results of 2 RCTs (29, 33) (*n* = 433) showed that the flavonoids group significantly decreased patients hospitalized and requiring mechanical ventilation compared with the control group (*P* = 0.986, *I*^2^ = 0%; RR = 0.31, 95% CI:0.12 to 0.77, *P* = 0.012).

8) Days of hospitalization

Pooled results of 2 RCTs (32, 33) (*n* = 276) showed that the flavonoids group significantly decreased days of hospitalization vs. the control group (*P* = 0.259, *I*^2^ = 21.6%; WMD = −5.09, 95% CI: −5.76 to −4.41, *P* < 0.001). Results of each subgroup analysis (inpatients or outpatients) showed that the flavonoids group significantly decreased days of hospitalization vs. the control group (WMD = −0.59, 95% CI: −0.97 to −0.21, *P* = 0.002; WMD = −2.35, 95% CI: −2.77 to −1.94, *P* < 0.001; respectively) (Supplementary Table 2).

9) Changes of laboratory test index (CRP, LDH, Ferritin, and D-dimer)

Three RCTs (29–31) (*n* = 679) reported changes of laboratory test index as outcomes, and small heterogeneity was observed. Pooled results showed that the flavonoids group significantly decreased the level of C-reactive protein (CRP) and lactate dehydrogenase (LDH) compared with the control group (WMD = −0.68, 95% CI: −0.86 to −0.48, *P* < 0.001; WMD = −50.83, 95% CI: −84.07 to −17.59, *P* = 0.003; respectively). Moreover, the flavonoids group significantly increased the level of D-dimer compared with the control group (WMD = 0.61, 95% CI:0.34 to 0.89, *P* < 0.001). However, the level of ferritin did not differ in the two groups (WMD = −89.48, 95% CI: −204.94 to 25.99, *P* = 0.129). Subgroup analyses were performed based on whether the patient was hospitalized (inpatients or outpatients), results showed that the flavonoids group significantly decreased changes of CRP vs. the control group in the inpatients' subgroup instead of outpatients' subgroup. Furthermore, the flavonoids group significantly decreased changes of LDH in both inpatients and outpatients' subgroups; the flavonoids group significantly increased changes of D-dimer in inpatients subgroup instead of outpatients' subgroup, and changes of ferritin did not differ between the two groups in any subgroup (inpatients or outpatients) (Supplementary Table 2).

10) Incidence of adverse reactions

All of the 4 RCTs (29–32) (*n* = 936) reported that no adverse effects related to both the flavonoids group and the control group were observed in participants (Supplementary Table 3). Thus, the flavonoids group did not increase the incidence of adverse reactions compared to the control group.

#### Acute Non-streptococcal Tonsillopharyngitis

Three RCTs (34–36) investigated the efficacy and safety of flavonoids in acute non-streptococcal tonsillopharyngitis.

(1) Result of the primary outcome: change of TSS on day 7

**Figure 5:**
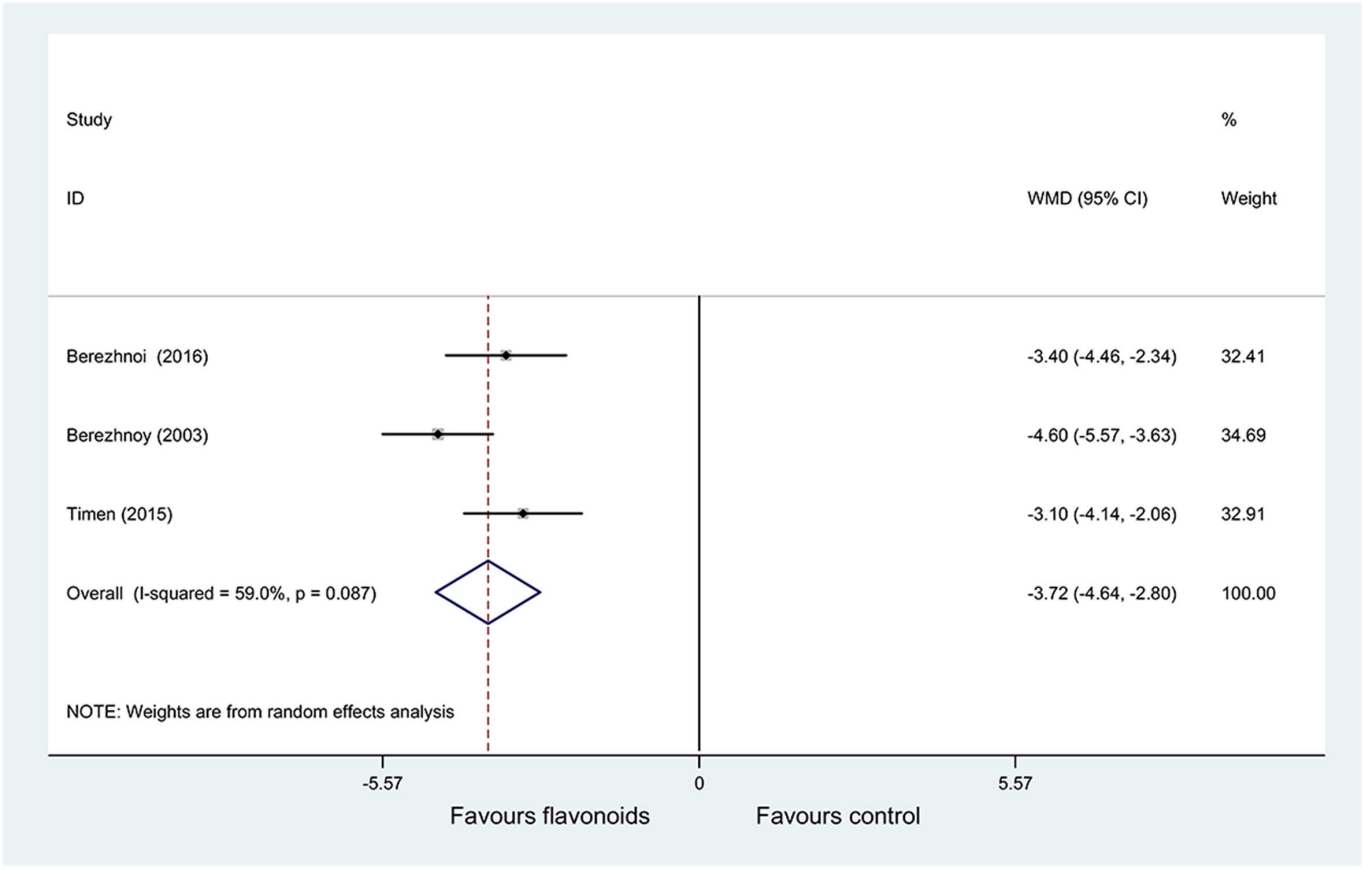
Meta-analysis of change of tonsillitis severity score (TSS) on day 7 in patients with acute non-streptococcal tonsillopharyngitis.

Pooled results of 3 RCTs (34–36) (*n* = 345) with a random-effects model showed that the flavonoids group significantly decreased the TSS on day 7 vs. the control group (*P* = 0.087, *I*^2^ = 59%; WMD = −3.72, 95% CI: −4.64 to −2.8, *P* < 0.001) (Figure 5). Subgroup analysis was performed based on sample size (> 140 or ≤ 140), the results of each subgroup analysis showed that the flavonoids group significantly decreased the TSS on day 7 compared with the control group with no heterogeneity (WMD = −4.6, 95% CI: −5.57 to −3.63, *P* < 0.001; WMD = −3.25, 95% CI: −3.99 to −2.51, *P* < 0.001; respectively) (Supplementary Table 1).

(2) Results of the secondary outcomes

As for secondary outcomes (Table 5), pooled results of 3 RCTs (34–36) with a fixed-effects model showed that the flavonoids group significantly improved major improved or completely recovered on the IMOS on day 4 compared with the control group (*P* = 0.141, *I*^2^ = 49.0%; RR = 4.01, 95% CI: 2.89 to 5.56, *P* < 0.001). Results also showed that the flavonoids group significantly increased the complete improvement rate of symptoms including fever, headache, difficulty in swallowing, sore throat, salivation, and pharyngeal erythema on day 4 vs. the control group (RR = 1.86, 95% CI: 1.53 to 2.25, *P* < 0.001; RR = 1.89, 95% CI: 1.4 to 2.55, *P* < 0.001; RR = 2.22, 95% CI: 1.45 to 3.4, *P* < 0.001; RR = 2.25, 95% CI: 1.65 to 3.05, *P* < 0.001; RR = 1.62, 95% CI: 1.37 to 1.92, *P* < 0.001; RR = 2.26, 95% CI: 1.49 to 3.43, *P* < 0.001; respectively). Moreover, the flavonoids group significantly decreased the incidence of patients unable to work after treatment and increased the IMPSS vs. the control group (RR = 0.24, 95% CI:0.15 to 0.4, *P* < 0.001; RR = 3.61, 95% CI: 2.37 to 5.51, *P* < 0.001; respectively). Pooled results of 3 RCTs (34–36) with 345 patients showed that the incidence of adverse reactions did not differ between the two groups (*P* = 0.018, *I*^2^ = 75%; RR = 0.318, 95% CI:0.08 to 1.29, *P* = 0.108).

**Table 5:** Results of the secondary outcomes in acute non-streptococcal tonsillopharyngitis.

**Outcomes**		**No. of trial**	**WMD/RR**	**95% CI**	***P***	***I^**2**^*; *P***	**Effect model**
Major improved or completely recovered on the IMOS on day 4		3	4.01	2.89 to 5.56	<0.001	49.0%; 0.141	FE
Complete improvement rate of symptoms on day 4	Fever	3	1.86	1.53 to 2.25	<0.001	0.0%; 0.392	FE
	Headache	3	1.89	1.40 to 2.55	<0.001	67.3%; 0.047	RE
	Difficulty in swallowing	1	2.22	1.45 to 3.40	<0.001	/	FE
	Sore throat	3	2.25	1.65 to 3.05	<0.001	0.0%; 0.588	FE
	Salivation	3	1.62	1.37 to 1.92	<0.001	0.0%; 0.917	FE
	Pharyngeal erythema	3	2.26	1.49 to 3.43	<0.001	0.0%; 0.576	FE
Incidence of patients unable to work after treatment		1	0.24	0.15 to 0.40	<0.001	/	FE
IMPSS		1	3.61	2.37 to 5.51	<0.001	/	FE
Incidence of adverse reactions		3	0.318	0.08 to 1.29	0.108	75.0%; 0.018	RE

*IMOS, integrative medicine outcome scale; IMPSS, integrative medicine patient satisfaction scale; FE, fixed-effects model; RE, random-effects model*.

#### Acute Rhinosinusitis

One RCT (37) investigated the efficacy and safety of flavonoids in acute rhinosinusitis. Results were shown in Table 6.

**Table 6:** Results of outcomes in acute rhinosinusitis and bronchial pneumonia.

**Outcomes**		**No. of trial**	**WMD/RR**	**95% CI**	***P***	***I^**2**^*; *P***	**Effect model**
**Acute rhinosinusitis: primary outcome**							
Change of the SSS on day 7		1	−3.00	−3.93 to −2.07	<0.001	/	FE
**Acute rhinosinusitis: secondary outcomes**							
Major improved or completely recovered on the IMOS on day 7		1	5.20	1.60 to 16.88	0.006	/	FE
Complete improvement rate of fever on day 7		1	1.20	1.03 to 1.41	0.023	/	FE
Radiographic cure on day 21		1	4.08	1.82 to 9.14	0.001	/	FE
IMPSS		1	2.46	1.63 to 3.72	<0.001	/	FE
Number of patients unable to work on day 7		1	0.59	0.39 to 0.89	0.011	/	FE
Days-off work and duration of illness		1	−7.20	−10.86 to −3.54	<0.001	/	FE
Incidence of adverse reactions		1	3.06	0.65 to 14.46	0.158	/	FE
**Bronchial pneumonia: primary outcome**							
Time to symptoms disappearance	Fever	1	−2.30	−2.39 to −2.21	<0.001	/	FE
	Cough	1	−1.90	−2.06 to −1.74	<0.001	/	FE
	Lung rale	1	−2.20	−2.58 to −1.82	<0.001	/	FE
**Bronchial pneumonia: secondary outcomes**							
IL−6		1	−18.10	−21.19 to −15.01	<0.001	/	FE
IL−8		1	−18.10	−21.01 to −15.19	<0.001	/	FE
TNF-α		1	−26.00	−29.80 to −22.20	<0.001	/	FE
IL−10		1	17.70	14.46 to 20.94	<0.001	/	FE
Incidence of complications		1	0.22	0.10 to 0.51	<0.001	/	FE
Total incidence of adverse reactions		1	0.18	0.10 to 0.33	<0.001	/	FE

*SSS, sinusitis severity score; IMOS, integrative medicine outcome scale; IMPSS, integrative medicine patient satisfaction scale; IL−6, interleukin−6; IL−8, interleukin−8; TNF-α, tumor necrosis factor-α; IL−10, interleukin−10; FE, fixed-effects model; RE, random-effects model*.

(1) Result of the primary outcome: change of the SSS on day 7

Result of 1 RCT (37) (*n* = 103) showed that the flavonoids group significantly decreased the SSS on day 7 compared with the control group (WMD = −3, 95% CI: −3.93 to −2.07, *P* < 0.001).

(2) Results of the secondary outcomes

As for secondary outcomes, results showed that the flavonoids group significantly increased the major improved or completely recovered on the IMOS on day 7, the complete improvement rate of fever on day 7, radiographic cure on day 21, and IMPSS vs. the control group (RR = 5.2, 95% CI: 1.6 to 16.88, *P* = 0.006; RR = 1.2, 95% CI: 1.03 to 1.41, *P* = 0.023; RR = 4.08, 95% CI: 1.82 to 9.14, *P* = 0.001; RR = 2.46, 95% CI: 1.63 to 3.72, *P* < 0.001; respectively). Furthermore, the flavonoids group significantly decreased the number of patients unable to work on day 7 and days-off work and duration of illness vs. the control group (RR = 0.59, 95% CI:0.39 to 0.89, *P* = 0.011; WMD = −7.2, 95% CI: −10.86 to −3.54, *P* < 0.001; respectively). However, the incidence of adverse reactions did not differ between the two groups (RR = 3.06, 95% CI:0.65 to 14.46, *P* = 0.158).

#### Acute Bronchitis

Seven RCTs (38–44) investigated the efficacy and safety of flavonoids in acute bronchitis.

(1) Result of the primary outcome: change of BSS on day 7

**Figure 6:**
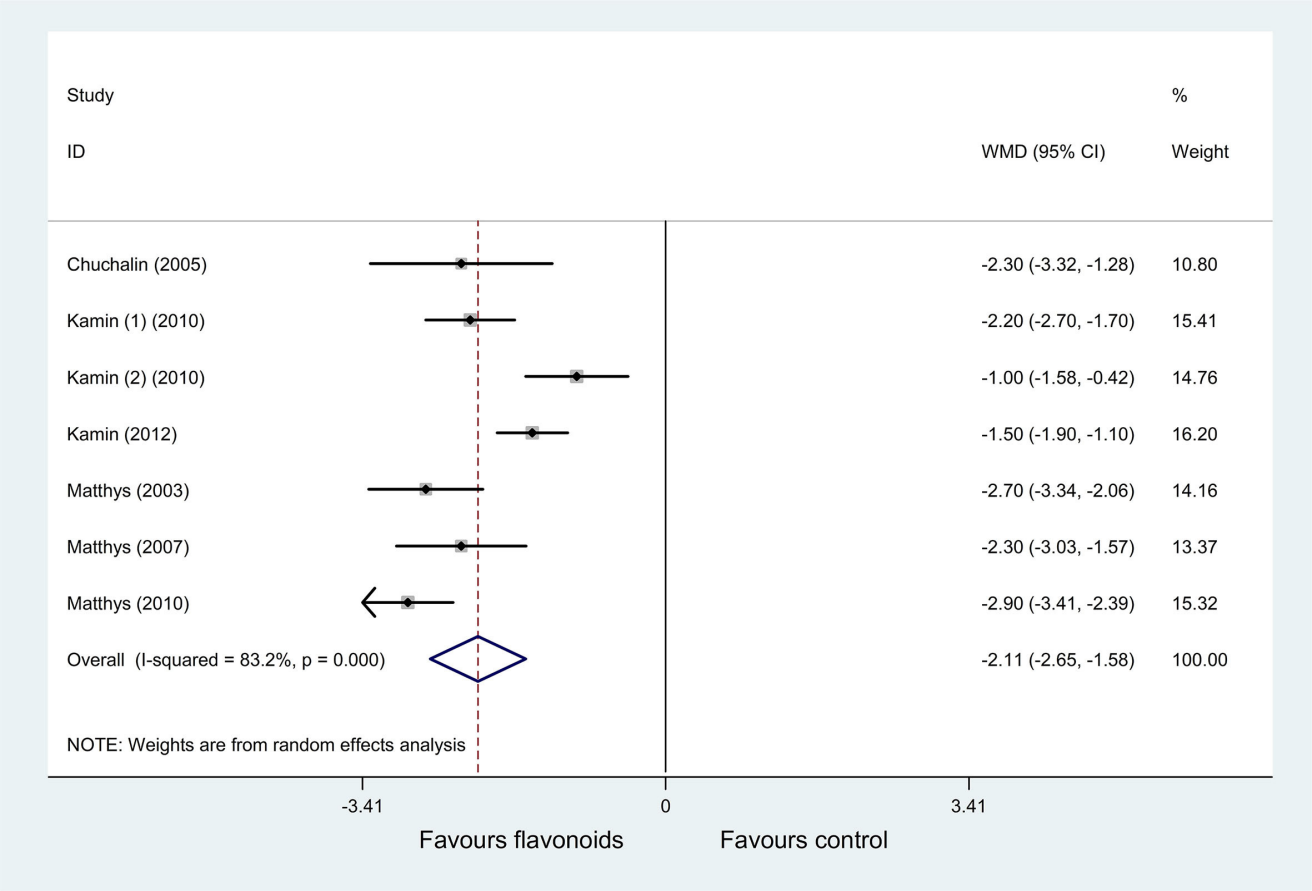
Meta-analysis of change of bronchitis severity score (BSS) on day 7 in patients with acute bronchitis.

Pooled results of 7 RCTs (38–44) (*n* = 2,031) with a random-effects model showed that the flavonoids group significantly decreased the BSS on day 7 compared with the control group (*P* < 0.001, *I*^2^ = 83.2%; WMD = −2.11, 95% CI: −2.65 to −1.58, *P* < 0.001) (Figure 6). Subgroup analysis was performed based on age (adults or children and adolescents). In each subgroup, results showed that the flavonoids group significantly decreased the BSS on day 7 vs. the control group (*I*^2^ = 0%, WMD = −2.66, 95% CI: −2.99 to −2.33, *P* < 0.001; *I*^2^ = 79.9%, WMD = −1.58, 95% CI: −2.2 to −0.95, *P* < 0.001; respectively) (Supplementary Table 1).

(2) Results of the secondary outcomes

As for secondary outcomes (Table 7), results showed that the flavonoids group significantly increased the major improved or completely recovered on the IMOS on day 7 and IMPSS vs. the control group (RR = 2.3, 95% CI: 1.63 to 3.26, *P* < 0.001; RR = 1.96, 95% CI: 1.64 to 2.34, *P* < 0.001; respectively). Furthermore, the flavonoids group significantly increased the complete improvement rate of symptoms including headache, coughing, sputum production, hoarseness, rales/rhonchi, and fatigue/exhaustion vs. the control group (RR = 1.29, 95% CI: 1.07 to 1.55, *P* = 0.006; RR = 3.22, 95% CI: 1.17 to 8.89, *P* = 0.024; RR = 1.71, 95% CI: 1.25 to 2.34, *P* = 0.001; RR = 1.63, 95% CI: 1.45 to 1.84, *P* < 0.001; RR = 1.77, 95% CI: 1.57 to 1.99, *P* < 0.001; RR = 1.48, 95% CI: 1.3 to 1.68, *P* < 0.001; respectively). However, the complete improvement rate of symptoms including fever, dyspnea, and pain in the limbs did not differ between the two groups. Moreover, the flavonoids group significantly decreased the number of patients unable to work on day 7 and days-off work and duration of illness vs. the control group (RR = 0.38, 95% CI:0.22 to 0.66, *P* = 0.001; WMD = −1.6, 95% CI: −2.35 to −0.85, *P* < 0.001; respectively). Pooled results of 7 RCT (38–44) with a fixed-effects model showed that the incidence of adverse reactions did not differ between the two groups (*P* = 0.891, *I*^2^ = 0%; RR = 1.23, 95% CI:0.96 to 1.58, *P* = 0.109).

**Table 7:** Results of the secondary outcomes in acute bronchitis.

**Outcomes**		**No. of trial**	**WMD/RR**	**95% CI**	***P***	***I^**2**^*; *P***	**Effect model**
Major improved or completely recovered on the IMOS on day 7		7	2.30	1.63 to 3.26	<0.001	90.4%; <0.001	RE
IMPSS		7	1.96	1.64 to 2.34	<0.001	72.7%; 0.001	RE
Complete improvement rate of symptoms	Headache	3	1.29	1.07 to 1.55	0.006	77.2%; 0.012	RE
	Coughing	3	3.22	1.17 to 8.89	0.024	91.2%; <0.001	RE
	Sputum production	3	1.71	1.25 to 2.34	0.001	67.8%; 0.045	RE
	Hoarseness	3	1.63	1.45 to 1.84	<0.001	36.0%; 0.209	FE
	Rales/rhonchi	3	1.77	1.57 to 1.99	<0.001	0.0%; 0.907	FE
	Fatigue/exhaustion	3	1.48	1.30 to 1.68	<0.001	43.4%; 0.171	FE
	Fever	3	1.24	0.91 to 1.70	0.180	95.3%; <0.001	RE
	Dyspnea	2	1.35	0.95 to 1.92	0.099	85.5%; 0.009	RE
	Pain in the limbs	3	1.24	0.97 to 1.58	0.088	91.5%; <0.001	RE
Number of patients unable to work on day 7		5	0.38	0.22 to 0.66	0.001	94.0%; <0.001	RE
Days-off work and duration of illness		1	−1.60	−2.35 to −0.85	<0.001	/	FE
Incidence of adverse reactions		7	1.23	0.96 to 1.58	0.109	0.0%; 0.891	FE

*IMOS, integrative medicine outcome scale; IMPSS, integrative medicine patient satisfaction scale; FE, fixed-effects model; RE, random-effects model*.

#### Bronchial Pneumonia

One RCT (45) investigated the efficacy and safety of flavonoids in bronchial pneumonia. Results were shown in Table 6.

(1) Result of the primary outcome: time to symptoms disappearance

Results of 1 RCT (45) (*n* = 180) showed that the flavonoids group significantly decreased the time to symptoms disappearance including fever, cough, and lung rale compared with the control group (WMD = −2.3, 95% CI: −2.39 to −2.21, *P* < 0.001; WMD = −1.9, 95% CI: −2.06 to −1.74, *P* < 0.001; WMD = −2.2, 95% CI: −2.58 to −1.82, *P* < 0.001; respectively).

(2) Results of the secondary outcomes

As for secondary outcomes, results showed that the flavonoids group significantly decreased the level of interleukin−6 (IL−6), interleukin−8 (IL−8), and tumor necrosis factor-α (TNF-α) compared with the control group (WMD = −18.1, 95% CI: −21.19 to −15.01, *P* < 0.001; WMD = −18.1, 95% CI: −21.01 to −15.19, *P* < 0.001; WMD = −26, 95% CI: −29.8 to −22.2, *P* < 0.001; respectively). However, the flavonoids group significantly increased the level of interleukin−10 (IL−10) vs. the control group (WMD = 17.7, 95% CI: 14.46 to 20.94, *P* < 0.001). Furthermore, the flavonoids group significantly decreased the incidence of complications and the total incidence of adverse reactions vs. the control group (RR = 0.22, 95% CI:0.1 to 0.51, *P* < 0.001; RR = 0.18, 95% CI:0.1 to 0.33, *P* < 0.001; respectively).

#### Upper Respiratory Tract Infections

Four eligible RCTs (46–49) investigated the efficacy and safety of flavonoids in upper respiratory tract infections.

(1) Results of the primary outcomes: change of total CIS on day 7 and the improvement rate of symptoms

**Figure 7:**
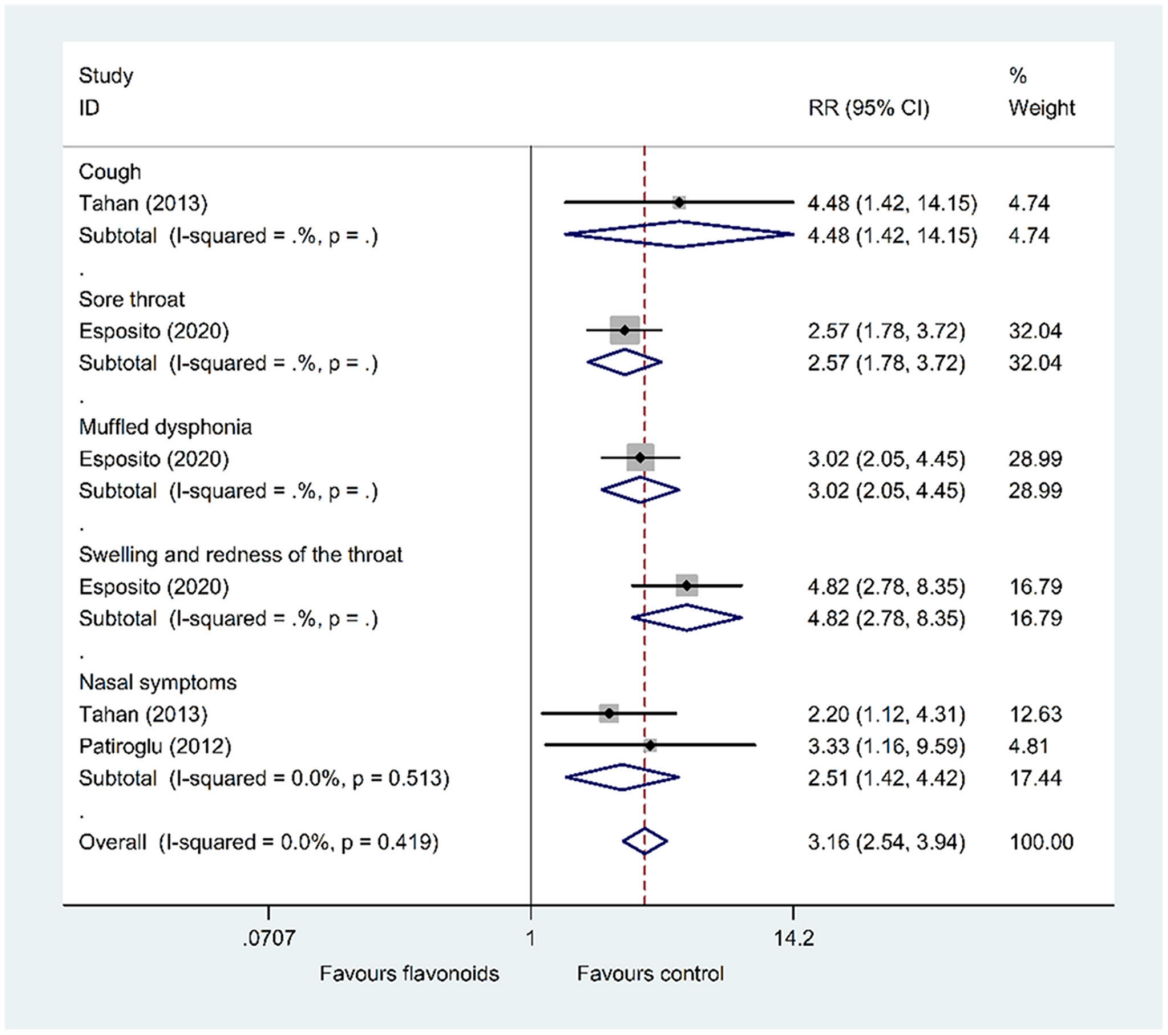
Meta-analysis of the improvement rate of symptoms in patients with upper respiratory tract infections.

Results of 1 RCT (47) (*n* = 160) showed that the flavonoids group significantly decreased the total CIS on day 7 compared with the control group (WMD = −7, 95% CI: −8.28 to −5.72, *P* < 0.001). Furthermore, pooled results of 3 RCTs (46, 48, 49) showed that the flavonoids group significantly increased the improvement rate of symptoms including cough, sore throat, muffled dysphonia, swelling and redness of the throat, and nasal symptoms compared with the control group (RR = 4.48, 95% CI: 1.42 to 14.15, *P* = 0.011; RR = 2.57, 95% CI: 1.78 to 3.72, *P* < 0.001; RR = 3.02, 95% CI: 2.05 to 4.45, *P* < 0.001; RR = 4.82, 95% CI: 2.78 to 8.35, *P* < 0.001; RR = 2.51, 95% CI: 1.42 to 4.42, *P* = 0.001; respectively) (Figure 7).

(2) Result of the secondary outcome

Two RCTs (46, 47) (*n* = 282) reported the incidence of adverse reactions. Pooled results showed that the incidence of adverse reactions did not differ between the two groups (RR = 1.46, 95% CI:0.72 to 2.94, *P* = 0.296).

#### Sensitivity Analysis

Results of sensitivity analysis on outcome indicators with obvious heterogeneity were shown in Supplementary Table 4.

(1) In common cold

Significant heterogeneity was observed among the included RCTs for outcomes of the total CIS (*I*^2^ = 82.5%), change of the sum of SSID of CIS (*I*^2^ = 67.6%), and major improvement or completely recovered according to the IMOS after treatment (*I*^2^ = 56.6%).

As for the change of the total CIS (*I*^2^ = 82.5%), Turner et al. (24) used troxerutin and Zn gluconate (flavonoids group) vs. Zn gluconate (control group) as interventions, however, other 4 RCTs used flavonoids vs. placebo as interventions. Thus, the heterogeneity may be caused by different intervention drugs. The sensitivity analysis result showed that after excluding Turner et al. (24), heterogeneity was decreased (*I*^2^ = 0%). Pooled results with a fixed-effects model showed that the flavonoids group still significantly decreased the total CIS vs. the control group (WMD = −4.13, 95% CI: −4.85 to −3.4, *P* < 0.001).

As for the change of the sum of SSID of CIS (*I*^2^ = 67.6%), the study conducted by Riley et al. (22) used taking capsules as a route of administration, however, other included RCTs used drinking. Furthermore, the intervention of the experimental group used in the study of Schutz et al. (23) was verum which was different from other included RCTs with EPs 7630. Thus, the heterogeneity may be caused by a different route of administration and types of intervention drugs. After excluding the above 2 RCTs, the heterogeneity decreased significantly (*I*^2^ = 0%), and the results still showed that the flavonoids group significantly decreased the sum of SSID of CIS vs. the control group (WMD = −7.3, 95% CI: −9.2 to −5.41, *P* < 0.001).

As for major improved or completely recovered according to the IMOS after treatment (*I*^2^ = 56.6%), after excluding the RCT published very early and conducted by Lizogub et al. (20), the remaining two RCTs were both conducted by David S. Riley and the heterogeneity decreased significantly (*I*^2^ = 0%), and the results still showed that the flavonoids group significantly increased the major improved or completely recovered patients according to the IMOS after treatment vs. the control group (RR = 3.85, 95% CI: 2.45 to 6.07, *P* < 0.001).

(2) In influenza

Significant heterogeneity was observed among the included RCTs for the outcome of the duration of fever (*I*^2^ = 94.5%). The two included RCTs (27, 28) were different in publication year (1995 vs. 2004), sample size (27 vs. 60), dosage (children received two and adults four tablespoons daily vs. 4 × 15 ml daily), and duration (3 days vs. 5 days). These aspects may be the sources of heterogeneity. Sensitivity analysis results with fixed-effects model showed that whether Zakay-Rones et al. (27) or (28) was excluded, the flavonoids group significantly decreased duration of fever compared with the control (WMD = −4, 95% CI: −5.01 to −2.99, *P* < 0.001; WMD = −0.97, 95% CI: −1.93 to −0.01, *P* = 0.048; respectively). However, the sensitivity analysis results were different from the pooled results of the two RCTs (27, 28) with the random-effects model (*P* >0.05). Thus, the pooled results for the outcome of the duration of fever should be treated with caution.

(3) In acute non-streptococcal tonsillopharyngitis

Significant heterogeneity was observed among the included RCTs for outcomes of the change of TSS on day 7 (*I*^2^ = 59%), the complete improvement rate of headache on day 4 (*I*^2^ = 67.3%), and incidence of adverse reactions (*I*^2^ = 75%).

As for the change of TSS on day 7 (*I*^2^ = 59%), the results of subgroup analysis have shown that heterogeneity may be related to sample size. As for the complete improvement rate of headache on day 4 (*I*^2^ = 67.3%), after excluding one RCT conducted by Berezhnoy et al. (35) published very early, the heterogeneity decreased significantly (*I*^2^ = 0%), and the results still showed that the flavonoids group significantly decreased the TSS on day 7 and increased complete improvement rate of headache on day 4 compared with the control group (WMD = −3.25, 95% CI: −3.99 to −2.51, *P* < 0.001; RR = 1.63, 95% CI: 1.34 to 1.98, *P* < 0.001; respectively).

As for the incidence of adverse reactions (*I*^2^ = 75%), in the study of Timen et al. (36), the dosage of EPs 7630 was 20 drops hourly on the first 2 days while awake and thereafter 3 × 20 drops daily for a further 4 days, however, the dosage of EPs 7630 was 3 × 20 drops daily in other included RCTs. Thus, the heterogeneity may be caused by the dosage of EPs 7630. After excluding Timen et al. (36), the heterogeneity decreased significantly (*I*^2^ = 33.4%), and the results with a fixed-effects model showed that the flavonoids group significantly decreased the incidence of adverse reactions vs. the control (RR = 0.17, 95% CI:0.07 to 0.43, *P* < 0.001). However, this sensitivity analysis result was different from the pooled result of the 3 RCTs (34–36) with the random-effects model (*P* >0.05), and the reason for the different results may be caused by the dosage of the drug. Thus, the pooled results for the outcome of incidence of adverse reactions should be treated with caution.

(4) In acute bronchitis

Significant heterogeneity was observed among the included RCTs for outcomes of the change of BSS on day 7 (*I*^2^ = 83.2%), major improved or completely recovered on the IMOS on day 7 (*I*^2^ = 90.4%), IMPSS (*I*^2^ = 72.7%), complete improvement rate of symptoms (headache, coughing, sputum production, fever, dyspnea, and pain in the limbs; *I*^2^ = 77.2%, 91.2%, 67.8%, 95.3%, 85.5%, and 91.5%, respectively), and the number of patients unable to work on day 7 (*I*^2^ = 94%).

As for the change of BSS on day 7 (*I*^2^ = 83.2%), participants were children and adolescents in 3 RCTs (39–41) and adults in other included RCTs. Thus, the heterogeneity may be caused by the age of participants. After excluding the above 3 RCTs (39–41), the heterogeneity decreased significantly (*I*^2^ = 0%), and the results with a fixed-effects model still showed that the flavonoids group significantly decreased the BSS on day 7 vs. the control group (WMD = −2.66, 95% CI: −2.99 to −2.33, *P* < 0.001).

As for major improvements or completely recovered on the IMOS on day 7 (*I*^2^ = 90.4%), sensitivity analysis results showed that regardless of which RCT was excluded separately, the heterogeneity was still obvious, and the direction of the outcome effect size remains unchanged. However, the pooled results for the outcome of major improved or completely recovered on the IMOS on day 7 should be treated with caution because of the significant heterogeneity.

As for IMPSS (*I*^2^ = 72.7%), after excluding 2 RCTs (39, 44), the heterogeneity decreased significantly (*I*^2^ = 0%), and the results with a fixed-effects model still showed that the flavonoids group significantly increased IMPSS vs. the control group (WMD = 1.7, 95% CI: 1.55 to 1.88, *P* < 0.001).

As for complete improvement rate of symptoms (headache, coughing, sputum production, fever, dyspnea, and pain in the limbs; *I*^2^ = 77.2%, 91.2%, 67.8%, 95.3%, 85.5%, and 91.5%, respectively), sensitivity analysis results showed that the pooled results of the outcomes of complete improvement rate of symptoms (headache, coughing, sputum production) were robust. However, as for the complete improvement rate of fever and pain in the limbs, the improvement of the complete improvement rate of fever and pain in the limbs was significantly greater in 1 RCT conducted by Matthys et al. (42) vs. the other two included RCTs (38, 43). After excluding Matthys et al. (42), the heterogeneity decreased significantly (*I*^2^ = 22%, 45.7%, respectively), and the results with a fixed-effects model showed that the flavonoids group significantly increased complete improvement rate of fever and pain in the limbs vs. the control group (WMD = 1.08, 95% CI: 1.01 to 1.15, *P* = 0.032; WMD = 1.11, 95% CI: 1.03 to 1.21, *P* = 0.01; respectively). Furthermore, two RCTs (38, 43) reported the outcome of complete improvement rate of dyspnea, the result of Chuchalin et al. (38) showed that the flavonoids group significantly increased the complete improvement rate of dyspnea vs. the control group (WMD = 1.63, 95% CI: 1.23 to 2.05, *P* < 0.001); however, the result of Matthys et al. (43) showed that although there is a trend of improvement, the complete improvement rate of dyspnea did not differ between the flavonoids group and the control group (WMD = 1.15, 95% CI:0.99 to 1.32, *P* = 0.056). The sensitivity analysis results of the complete improvement rate of symptoms (fever, dyspnea, pain in the limbs) differed from the previous pooled result with the random-effects model (*P* >0.05). Thus, the pooled results for the outcome of complete improvement rate of symptoms (fever, dyspnea, pain in the limbs) should be treated with caution.

As for the number of patients unable to work on day 7 (*I*^2^ = 94%), sensitivity analysis results showed that regardless of which RCT was excluded separately, the heterogeneity was still obvious, and the direction of the outcome effect size remains unchanged. Thus, the pooled results for the outcome of the number of patients unable to work on day 7 should be treated with caution because of the significant heterogeneity.

#### Publication Bias

**Figure 8:**
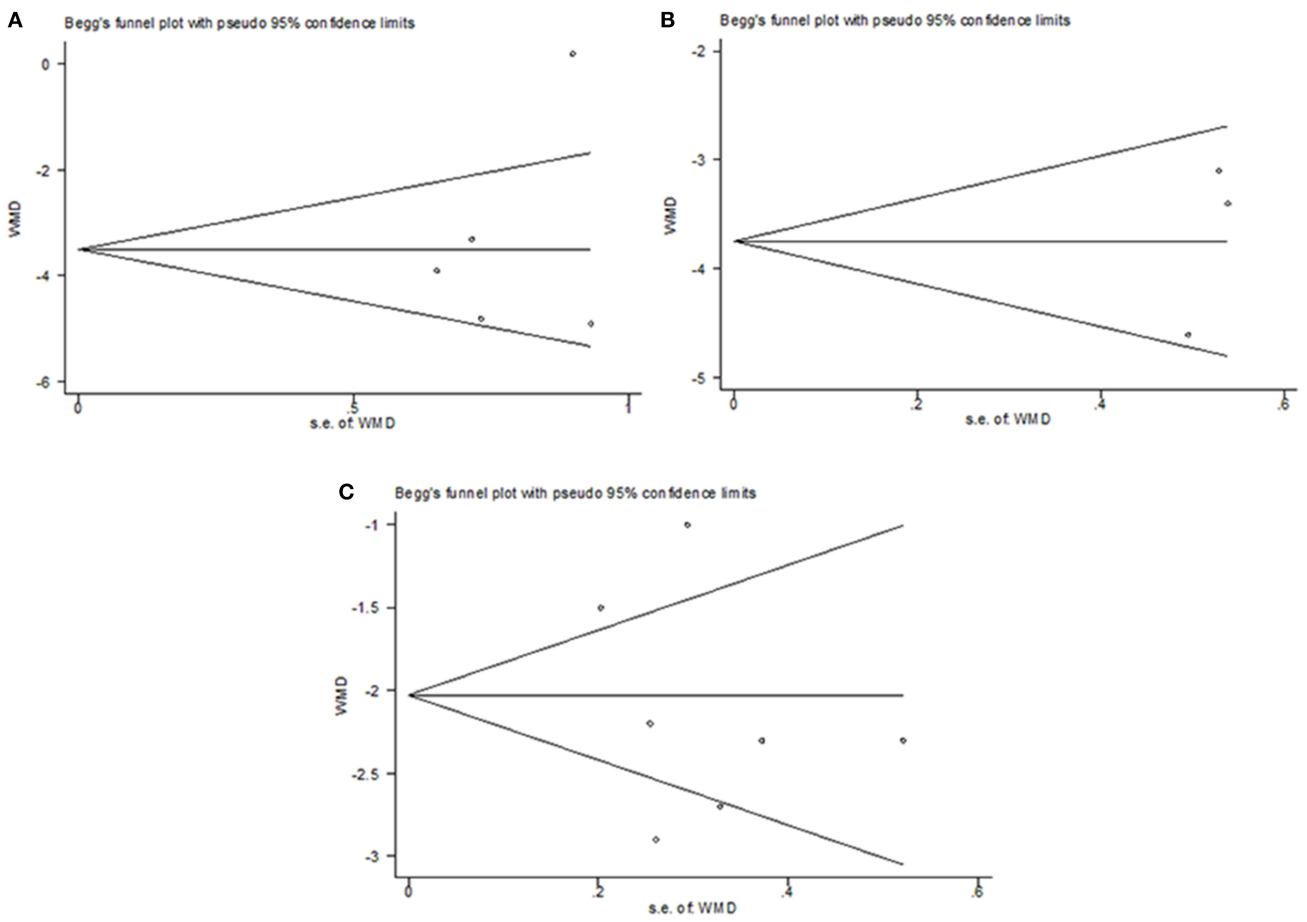
Publication bias analysis. **(A)** change of the total CIS in common cold, **(B)** change of TSS on day 7 in acute non-streptococcal tonsillopharyngitis, **(C)** and change of BSS on day 7 in acute bronchitis.

Publication bias analysis was conducted on the outcome of the change of the total CIS in common cold, change of TSS on day 7 in acute non-streptococcal tonsillopharyngitis, and change of BSS on day 7 in acute bronchitis. The funnel plots were symmetrical, most scatter points were inside the confidence limit. The *p*-value of Begg's tests were all 1, the *p*-value of Egger's tests were 0.558, 0.246, and 0.507, respectively. As shown in Figure 8, each of them did not show significant publication bias. However, because of the limited number of included RCTs in the present study, publication bias wasn't evaluated in the outcomes of influenza, COVID−19, acute rhinosinusitis, bronchial pneumonia, and upper respiratory tract infections.

## Discussion

Viral ARTIs are considered a common and public health problem. They affect people worldwide, even causing deaths, while the treatment options and curative effects are limited, such as typically symptom-related treatment, antivirals, palliative measures, etc. In recent years, the research of antiviral effects of flavonoids has received increasing attention. Based on a total of 30 RCTs, this systematic review and meta-analysis summarized the available evidence to show that flavonoids were efficacious and safe in the treatment of viral ARTIs including the common cold, influenza, COVID−19, acute non-streptococcal tonsillopharyngitis, acute rhinosinusitis, acute bronchitis, bronchial pneumonia, and upper respiratory tract infections. This study provided an initial set of evidence for potentially recommending flavonoids as a treatment option for viral ARTIs.

In common cold, results showed that the flavonoids group decreased the total CIS, the sum of SSID of CIS, and duration of inability to work vs. the control group; meanwhile, the flavonoids group improved clinical cure rate, the major improved or completely recovered according to the IMOS, the remission and improvement rates of symptoms, and IMPSS. In influenza, the flavonoids group improved the VAS for symptoms and complete cure on day 3 vs. the control group. In COVID−19, the flavonoids group decreased the time taken for alleviation of symptoms, time taken for SARS-CoV−2 RT-PCR clearance, the RT-PCR positive subjects at day 7, the median CGS at day 6, time to achievement of the normal status of symptoms, patients needed oxygen, patients hospitalized and requiring mechanical ventilation, patients in ICU, days of hospitalization, and mortality vs. the control group. Meanwhile, the flavonoids group increased the healed of symptoms on day 7, and patients were not hospitalized with the resumption of normal activities on day 6. In acute non-streptococcal tonsillopharyngitis, the flavonoids group decreased the TSS on day 7 and the incidence of patients unable to work. Meanwhile, the flavonoids group improved the major improvement or completely recovered on the IMOS on day 4, the complete improvement rate of symptoms on day 4, and IMPSS. In acute rhinosinusitis, the flavonoids group decreased the SSS on day 7, the number of patients unable to work on day 7, and days-off work and duration of illness vs. the control group. Meanwhile, the flavonoids group increased the major improved or completely recovered on the IMOS on day 7, the complete improvement rate of fever on day 7, radiographic cure on day 21, and IMPSS. In acute bronchitis, the flavonoids group decreased the BSS on day 7, the number of patients unable to work on day 7, and days-off work and duration of illness vs. the control group. Meanwhile, the flavonoids group increased the major improved or completely recovered on the IMOS on day 7 and IMPSS, and the complete improvement rate of symptoms. In bronchial pneumonia, the flavonoids group decreased the time to symptoms disappearance, the level of IL−6, IL−8, and TNF-α vs. the control group. In upper respiratory tract infections, the flavonoids group decreased total CIS on day 7 and increased the improvement rate of symptoms compared with the control group. Furthermore, results of the incidence of adverse reactions did not differ between the flavonoids and the control group, indicating that flavonoids were safe and well-tolerated in clinical trials and under the general usage conditions in clinical practice alike; therefore, members of society could take flavonoids supplements with reasonable confidence that the flavonoids component will not result in increased adverse events.

However, there was significant heterogeneity among the included studies for some outcomes. With the heterogeneity, we noted that the eligible trials varied in several respects, including differences in the study population, baseline comorbidities, intervention drugs, and methodological differences, which may contribute to substantial heterogeneity. We conducted sensitivity analyses and subgroup analyses to explore the heterogeneity. In common cold, for the outcome of the change of the total CIS in common cold, the substantial heterogeneity may be related to different interventions in the included RCTs; as for the change of the sum of SSID of CIS in common cold, the heterogeneity may be caused by a different route of administration and types of intervention drugs; as for major improved or completely recovered according to the IMOS after treatment in common cold, the heterogeneity may be caused by publication year and different researchers. In influenza, the heterogeneity may be caused by publication year, sample size, dosage, and duration. Furthermore, in COVID−19, for the outcome of time taken for alleviation of symptoms, median CGS at day 6, and time to achievement of the normal status of myalgia, the substantial heterogeneity may be related to the severity of COVID−19 (moderate or severe cases) in the included RCTs. In acute non-streptococcal tonsillopharyngitis, the heterogeneity may be related to the sample size, publication year, dosage of the flavonoids corresponding to the outcome of the change of the TSS on day 7, the complete improvement rate of headache on day 4, and the incidence of adverse reactions. In acute bronchitis, the heterogeneity may be caused by the age of participants in the outcome of the change of BSS on day 7. Sensitivity analysis results indicated that the most pooled results of these outcome indicators were robust. However, the following results of outcome indicators should be treated with caution because of instability and significant heterogeneity. In the outcome of the duration of fever in patients with influenza, the incidence of adverse reactions in patients with acute non-streptococcal tonsillopharyngitis, and complete improvement rate of symptoms (fever, dyspnea, pain in the limbs) in patients with acute bronchitis, results of sensitivity analysis were different from the previous pooled result with random-effects models. Furthermore, in the outcomes of major improved or completely recovered on the IMOS on day 7 and number of patients unable to work on day 7 in patients with acute bronchitis, sensitivity analysis results showed that regardless of which RCT was excluded separately, the heterogeneity was still obvious, and the direction of the outcome effect size remains unchanged.

To date, some systematic reviews and meta-analyses of RCTs investigating the efficacy of flavonoids in infectious respiratory diseases as well as some further reviews without formal meta-analyses have been published although, unlike our work, none of them considered all kinds of flavonoids in treating different viral ARTIs. A systematic review and meta-analysis conducted by Somerville et al. (18) mainly focused on the efficacy of flavonoids in preventing URTIs in healthy adults, results showed flavonoid supplementation decreased URTI incidence by 33% compared with control. Furthermore, 3 systematic reviews and meta-analyses (50–52) were conducted to investigate the efficacy of only pelargonium sidoides extract EPs 7630 in ARTIs. Careddu et al. (50) included 8 RCTs to investigate the application of EPs 7630 in acute bronchitis, acute tonsillopharyngitis, and ARTIs in children; results showed significant improvements of ARTIs symptom severity for EPs 7630 as compared to controls. Matthys et al. (51) included 13 trials and showed that EPs 7630 is an efficacious, safe, and well-tolerated herbal medicine in the management of ARTIs only including acute bronchitis, acute rhinosinusitis, and acute tonsillopharyngitis in children, adolescents, and adults. Agbabiaka et al. (52) only included 4 RCTs, and pooled results suggested that EPs7630 significantly reduced bronchitis symptom scores in patients with acute bronchitis on day 7. Furthermore, a review conducted by Wopker et al. (53) assessed complementary and alternative medicine including flavonoids in the treatment of acute bronchitis in children; results of the included studies indicated a favorable effect of investigated complementary and alternative medicine approaches; however, only three of 18 studies were RCTs, so Wopker et al. (53) concluded that a reliable statement on effectiveness was not possible and further RCTs are indispensable. In addition, a systematic review conducted by Wieland et al. (54) only included 5 randomized trials on elderberry for the treatment or prevention of viral respiratory illness and concluded that elderberry may be a safe option for treating viral respiratory illness; however, the evidence on both benefits and harms is uncertain and information from more studies is necessary to make firm conclusions. Moreover, 5 reviews were conducted to mainly investigate the mechanism of flavonoids against coronavirus infection (8, 55–58), some other reviews investigated some specific types of flavonoids including bee honey, propolis, ginkgo biloba, quercetin, naringenin, green tea, et al. against COVID−19 (17, 59–65). However, the above reviews mainly focused on the mechanism of flavonoids against coronavirus infection. The downside is that there is still a lack of a meta-analysis of RCTs to assess the efficacy and safety of flavonoids against COVID−19.

This systematic review and meta-analysis summarized the available evidence and showed that flavonoids were efficacious and safe in the treatment of viral ARTIs. Flavonoids are proposed to treat viral ARTIs because they have a range of physiologic effects in humans, including antiviral, anti-inflammatory, cytotoxic, antimicrobial, and antioxidant (13). Studies report flavonoids to have both an antiproliferative and anti-replicative effect on 2 common viral sources of viral ARTIs and reduce inflammation by decreasing NF-kB (66–68). These mechanisms, and others, may have the potential to treat viral ARTIs, which makes flavonoids a current field of interest in human immunity.

Specifically, the efficacy of flavonoids including EPs 7630, verum, and troxerutin in the common cold were investigated in this meta-analysis. Furthermore, *in vitro* evaluations have demonstrated the antiviral effects (69), direct and indirect antibacterial activity, as well as immunomodulatory capabilities of these flavonoids. As for the mechanism of flavonoids including lozenge, echinaforce hotdrink, and sambucol in treating influenza, the neuraminidase enzyme is essential for the influenza virus replication cycle (70), previous studies have proposed that flavonoids may bind to neuraminidase and inhibit its activity though no direct evidence exists (27, 71). A more recent study identified that flavonoids can bind to the surface of the H1N1 influenza virus and inhibit viral infection *in vitro* by interfering with host cell receptor recognition and/or blocking receptor binding (72). The efficacy of flavonoids including honey, nigella sativa, quercetin, and propolis in treating COVID−19 was investigated in this meta-analysis. Studies showed that flavonoids act via direct antiviral properties, where they inhibit different stages of the virus infective cycle and indirect effects when they modulate host responses to viral infection and subsequent complications. Flavonoids have shown antiviral activity via inhibition of viral protease, RNA polymerase, mRNA, virus replication, and infectivity. The compounds were also effective for the regulation of interferons, pro-inflammatory cytokines, and sub-cellular inflammatory pathways such as nuclear factor-κB and Jun N-terminal kinases (56). Furthermore, flavonoids can counteract the virus-mediated elevated levels of inflammatory cytokines leading to multiple organ failure (55, 57). Efficacy of flavonoids including EPs 7630 in treating acute non-streptococcal tonsillopharyngitis, acute rhinosinusitis, and acute bronchitis was investigated in 11 included RCTs of this meta-analysis. EPs 7630, one kind of flavonoid, has antiviral, antibacterial, and immunomodulatory activities (73, 74). Additionally, EPs 7630 improves silier activities (75). The effect of EPs 7630 in infectious conditions could also be explained by stimulation of the nonspecific immune system (76). EPs 7630 also mediates anti-infective activities by TLR signaling (77). Moreover, EPs 7630 was found to be a potent HIV−1 attachment inhibitor (78) and to interfere with the replication of seasonal influenza A virus strains, respiratory syncytial virus, human coronavirus, parainfluenza virus, and coxsackie virus (69). Anti-influenza virus activity of the herbal extract was also confirmed in an animal model (73). These antiviral effects may contribute to the efficacy of EPs 7630 in ARTIs. The efficacy of flavonoids including naringenin in bronchial pneumonia was investigated in this meta-analysis. Naringenin has been widely used for its various pharmacological potentials, such as anti-diabetic, anti-inflammatory, anti-oxidant, and immunomodulatory capacities (79). Manchope et al. found that naringenin activated Nrf2 in macrophages, inducing an anti-oxidative reaction (80). Felipe et al. reported that naringenin could inhibit NF- kB activation and reduce the production of pro-inflammatory cytokines such as IL−1 and TNF-a; thus, alleviating some inflammatory responses (81). Furthermore, Lawrence et al. (82) reported that naringenin prevented degradation of IkB *in vitro*, which is the endogenous inhibitor of NF- kB p50/p65 subunits. Whereas, when IkB degradation is inhibited, NF- kB transcription activity is also repressed; thus, relieving the excessive inflammation responses. These effects may contribute to the efficacy of naringenin in bronchial pneumonia. Efficacy of flavonoids including propolis, cistus incanus, and EPs 7630 on upper respiratory tract infections was investigated in 4 included RCTs of this meta-analysis. Propolis resulted to exert antioxidant and anti-inflammatory activities (83) through an epigenetic mechanism of action, modifying the expression level of microRNAs and mRNA targets coding for antioxidant enzymes and pro-inflammatory cytokines. Studies also showed that in experimental animals, the oral administration of propolis is followed by the rapid absorption and metabolism of galangin and the induced adaptation of the antioxidant first-line defense system (84). Furthermore, cistus incanus exhibited a wide range of antibacterial, antifungal, anti-inflammatory effects and have been shown to be strong antioxidants with potential health benefits.

However, there are also limitations of the current analysis that should be taken into consideration. Firstly, because of the limited number of included RCTs in the present study, publication bias wasn't evaluated in the outcomes of influenza, COVID−19, acute rhinosinusitis, bronchial pneumonia, and upper respiratory tract infections. Secondly, some RCTs were of poor quality, for example, were single-center with a few participants. Thirdly, there was significant heterogeneity among the included studies for some outcomes which may result from the differences in trial populations, treatment regimens, and methodological differences, especially for the pooled results of outcomes of the duration of fever in patients with influenza, the incidence of adverse reactions in patients with acute non-streptococcal tonsillopharyngitis, complete improvement rate of symptoms (fever, dyspnea, pain in the limbs) in patients with acute bronchitis, major improved or completely recovered on the IMOS on day 7 in patients with acute bronchitis, and the number of patients unable to work on day 7 in patients with acute bronchitis which should be treated with caution because of instability and significant heterogeneity. Fourthly, different platforms/analyses used in the study (RT-PCR for COVID−19), we failed to conduct subgroup analysis based on different regions and different platforms/analyses because of the limited RCTs. Given the limitations of the present study, we suggest that the conclusions need to be established or confirmed on a larger scale with more detailed instructions in future studies, especially in RCTs.

Viral ARTIs are considered a common and public health problem, while the treatment options and curative effects are limited. Flavonoids are proposed to treat viral ARTIs because they have a range of physiologic effects in humans, including antiviral, anti-inflammatory, cytotoxic, antimicrobial, and antioxidant. This systematic review and meta-analysis summarized the available evidence and showed that flavonoids were efficacious and safe in the treatment of viral ARTIs. This study provided an initial set of evidence for potentially recommending flavonoids as a treatment option for viral ARTIs. Two clear moderators of the effect of flavonoids on ARTIs are the dose and type, which also contribute to the practical application of a flavonoid intervention; currently, flavonoids used frequently in the treatment of ARTIs including EPs 7630, propolis, quercetin, naringenin, and troxerutin; thus, further research is needed to quantify the optimal type and dose of flavonoids, this information is meaningful because it describes the optimal prescription of flavonoid intake to treat ARTIs. Moreover, more detailed investigations on pharmacological mechanisms, long-term toxicology, bioavailability, as well as studies on the mechanism of flavonoids in treating ARTIs are required.

## Conclusion

Taken together, this systematic review and meta-analysis summarized the available evidence and showed that flavonoids were efficacious and safe in treating viral ARTIs including the common cold, influenza, COVID−19, acute non-streptococcal tonsillopharyngitis, acute rhinosinusitis, acute bronchitis, bronchial pneumonia, and upper respiratory tract infections. This study provided an initial set of evidence for potentially recommending flavonoids as a treatment option for viral ARTIs. However, uncertainty remains because there were few RCTs per type of ARTI and many of the RCTs were small and of low quality with a substantial risk of bias. Given the limitations of the present study, we suggest that the conclusions need to be established or confirmed on a larger scale with more detailed instructions in future studies, especially in RCTs.

## Data Availability Statement

The datasets presented in this study can be found in online repositories. The names of the repository/repositories and accession number(s) can be found in the article/Supplementary Material.

## Author Contributions

JY and G-JF: conceptualization. JY, YZ, and X-ZW: data curation. JZ: formal analysis. Z-JY, Y-PL, and LS: project administration. G-JF: supervision and writing—review and editing. JY, Q-YL, and G-JF: validation. JY and YZ: writing—original draft. All the authors have read and approved the manuscript.

## Funding

This study was partially supported by the Chinese Government, Ministry of Science, and Technology of the People's Republic of China through the National Science and Technology Support Program (Grant No. 2015BAI04B09) and Guangdong Provincial Hospital of Traditional Chinese Medicine (Grant No. 2021DB02).

## Conflict of Interest

The authors declare that the research was conducted in the absence of any commercial or financial relationships that could be construed as a potential conflict of interest.

## Publisher's Note

All claims expressed in this article are solely those of the authors and do not necessarily represent those of their affiliated organizations, or those of the publisher, the editors and the reviewers. Any product that may be evaluated in this article, or claim that may be made by its manufacturer, is not guaranteed or endorsed by the publisher.

## Abbreviations

ARTIs: acute respiratory tract infections
RCTs: randomized controlled trials
CIS: cold intensity score
SSID: sum of symptom intensity differences
TSS: tonsillitis severity score
SSS: sinusitis severity score
BSS: bronchitis severity score
IL-6: interleukin-6
IL-8: interleukin-8
TNF-α: tumor necrosis factor-α
ARTI: acute respiratory tract infection
URTI: upper respiratory tract infection
SARS-CoV-2: severe acute respiratory syndrome coronavirus 2
RCTs: randomized controlled trials
CIS: cold intensity score
SSID: sum of symptom intensity differences
IMOS: integrative medicine outcome scale
IMPSS: integrative medicine patient satisfaction scale
VAS: visual analog scores
CGS: clinical grading score
TSS: tonsillitis severity score
SSS: sinusitis severity score
BSS: bronchitis severity score
WMD: weighted mean difference
CI: confidence intervals
RR: relative risk
CRP: C-reactive protein
LDH: lactate dehydrogenase
IL-6: interleukin-6
IL-8: interleukin-8
TNF-α: tumor necrosis factor-α
IL-10: interleukin-10.
