# Supplementary material for: Flavonoids for Treating Viral Acute Respiratory Tract Infections: A Systematic Review and Meta-Analysis of 30 Randomized Controlled Trials
Source: Front Public Health. 2022 Feb 16;10:814669. doi: 10.3389/fpubh.2022.814669 (PMC8888526; doi:10.3389/fpubh.2022.814669)
Supplement: Supplementary file 1 [file Data_Sheet_1.docx]

**Supplementary Material**

**Supplementary Table 1. Results of subgroup analysis in outcomes with obvious heterogeneity**

| Outcomes | | No. of trial | WMD/RR | 95% CI | *P* | *I^2^; P* | Effect model |
| --- | --- | --- | --- | --- | --- | --- | --- |
| **COVID-19** | | | | | | | |
| Time taken for alleviation of symptoms | moderate cases | 1 | -3.70 | -3.90 to -3.50 | < 0.001 | / | FE |
|  | severe cases | 1 | -6.30 | -7.60 to -5.00 | < 0.001 | / | FE |
| CGS on day 6 | moderate cases | 1 | -1.00 | -1.20 to -0.80 | < 0.001 | / | FE |
|  | severe cases | 1 | -2.21 | -2.68 to -1.74 | < 0.001 | / | FE |
| Time to achievement of normal status of myalgia | moderate cases | 1 | -2.00 | -2.71 to -1.29 | < 0.001 | / | FE |
|  | severe cases | 1 | -4.00 | -6.19 to -1.82 | < 0.001 | / | FE |
| **Acute non-streptococcal tonsillopharyngitis** | | | | | | | |
| Change of TSS on day 7 | sample size > 140 | 1 | -4.60 | -5.57 to -3.63 | < 0.001 | / | FE |
|  | sample size ≤ 140 | 2 | -3.25 | -3.99 to -2.51 | < 0.001 | 0.0%; 0.691 | FE |
| **Acute bronchitis** | | | | | | | |
| Change of BSS on day 7 | adults | 4 | -2.66 | -2.99 to -2.33 | < 0.001 | 0.0%; 0.517 | FE |
|  | children and adolescents | 3 | -1.58 | -2.20 to -0.95 | < 0.001 | 79.9%; 0.007 | RE |

**Supplementary Table 2. Results of subgroup analysis based on inpatients or outpatients in COVID-19**

| Outcomes | | No. of trial | WMD/RR | 95% CI | *P* | *I^2^; P* | Effect model |
| --- | --- | --- | --- | --- | --- | --- | --- |
| Patients in ICU | inpatients | 1 | 0.42 | 0.19 to 0.93 | 0.033 | / | FE |
|  | outpatients | 2 | 0.10 | 0.01 to 0.77 | 0.027 | 0.0%; 0.404 | FE |
| Mortality | inpatients | 2 | 0.34 | 0.12 to 0.99 | 0.048 | 0.0%; 0.379 | FE |
|  | outpatients | 2 | 0.2 | 0.02 to 1.68 | 0.138 | 0.0%; 0.697 | FE |
| Patients needed oxygen | inpatients | 1 | 0.27 | 0.12 to 0.63 | 0.002 | 0.0%; 0.877 | FE |
|  | outpatients | 1 | 0.07 | 0.01 to 0.49 | 0.008 | / | FE |
| Days of hospitalization | inpatients | 1 | -0.59 | -0.97 to -0.21 | 0.002 | / | FE |
|  | outpatients | 1 | -2.35 | -2.77 to -1.94 | < 0.001 | / | FE |
| Changes of CRP | inpatients | 2 | -0.75 | -0.95 to -0.54 | < 0.001 | 0.0%; 0.458 | FE |
|  | outpatients | 1 | -1.01 | -0.71 to 0.50 | 0.743 | / | FE |
| Changes of LDH | inpatients | 1 | -41.82 | -77.45 to -6.19 | 0.021 | / | FE |
|  | outpatients | 1 | -111.30 | -203.62 to 18.98 | 0.018 | / | FE |
| Changes of D-dimer | inpatients | 1 | 0.64 | 0.34 to 0.94 | < 0.001 | / | FE |
|  | outpatients | 1 | 0.47 | -0.14 to 1.09 | 0.130 | / | FE |
| Changes of Ferritin | inpatients | 1 | -90.30 | -212.57 to 31.97 | 0.148 | / | FE |
|  | outpatients | 1 | -82.70 | -433.79 to 268.39 | 0.644 | / | FE |

**Supplementary Table 3. The original data extracted from the included 4 RCTs on adverse events in patients with COVID-19**

| Author, Year | Flavonoids group | | Control group | |
| --- | --- | --- | --- | --- |
|  | Adverse events | No adverse events | Adverse events | No adverse events |
| Ashraf 2020 (29) | 0 | 157 | 0 | 156 |
| Önal 2021 (30) | 0 | 49 | 0 | 380 |
| Pierro (1) 2021 (31) | 0 | 21 | 0 | 21 |
| Pierro (2) 2021 (32) | 0 | 76 | 0 | 76 |

**Supplementary Table 4. Results of sensitivity analysis on outcome indicators with high heterogeneity**

| Outcome | Excluded RCT | Remaining RCTs | WMD /RR | 95% CI | *P* | *I^2^; P* | Effect model |
| --- | --- | --- | --- | --- | --- | --- | --- |
| **Common cold** | | | | | | | |
| Total CIS | Turner et al. (24) | 4 | -4.13 | -4.85 to -3.40 | < 0.001 | 0.0%; 0.393 | FE |
| Change of the sum of SSID of CIS | Riley (2019) (22); Schu¨tz (2010) (23) | 2 | -7.30 | -9.20 to -5.41 | < 0.001 | 0.0%; 0.720 | FE |
| Major improved or completely recovered according to the IMOS | Lizogub (2007) (20) | 2 | 3.85 | 2.45 to 6.07 | < 0.001 | 0.0%; 0.654 | FE |
| **In influenza** | | | | | | | |
| Duration of fever | Zakay-Rones (1995) (27) | 1 | -4.00 | -5.01 to -2.99 | < 0.001 | / | FE |
|  | Zakay-Rones (2004) (28) | 1 | -0.97 | -1.93 to -0.01 | 0.048 | / | FE |
| **Acute non-streptococcal tonsillopharyngitis** | | | | | | | |
| Complete improvement rate of headache on day 4 | Berezhnoy (2003) (35) | 2 | 1.63 | 1.34 to 1.98 | < 0.001 | 0.0%; 0.547 | FE |
| Incidence of adverse reactions | Timen (2015) (36) | 2 | 0.17 | 0.07 to 0.43 | < 0.001 | 33.4%; 0.220 | FE |
| **Acute bronchitis** | | | | | | | |
| change of BSS on day 7 | Kamin(1) (2010) (39); Kamin(2) (2010) (40) Kamin (2012) (41) | 4 | -2.66 | -2.99 to -2.33 | < 0.001 | 91.3%; < 0.001 | FE |
| Major improved or completely recovered on the IMOS on day 7 | Chuchalin (2005) (38) | 6 | 2.23 | 1.54 to 3.25 | < 0.001 | 88.7%; < 0.001 | RE |
|  | Kamin(1) (2010) (39) | 6 | 2.10 | 1.51 to 2.92 | < 0.001 | 92.3%; < 0.0001 | RE |
|  | Kamin(2) (2010) (40) | 6 | 2.52 | 1.62 to 3.91 | < 0.001 | 92.0%; < 0.001 | RE |
|  | Kamin (2012) (41) | 6 | 2.36 | 1.54 to 3.61 | < 0.001 | 92.4%; < 0.001 | RE |
|  | Matthys (2003) (42) | 6 | 2.46 | 1.62 to 3.73 | < 0.001 | 91.3%; < 0.001 | RE |
|  | Matthys (2007) (43) | 6 | 2.53 | 1.77 to 3.62 | < 0.001 | 85.8%; < 0.001 | RE |
|  | Matthys (2010) (44) | 6 | 2.02 | 1.51 to 2.72 | < 0.001 | 86.6%; < 0.001 | RE |
| IMPSS | Kamin (1) (2010) (39); Matthys (2010) (44) | 5 | 1.70 | 1.55 to 1.88 | < 0.001 | 0.0%; 0.786 | FE |
| Complete improvement rate of headache | Chuchalin (2005) (38) | 2 | 1.42 | 1.29 to 1.58 | < 0.001 | 40.8%; 0.164 | FE |
| Complete improvement rate of coughing | Matthys (2003) (42) | 2 | 4.72 | 2.88 to 7.72 | < 0.001 | 0.0%; 0.576 | FE |
| Complete improvement rate of sputum production | Chuchalin (2005) (38) | 2 | 1.47 | 1.26 to 1.73 | < 0.001 | 27.4%; 0.240 | FE |
| Complete improvement rate of fever | Matthys (2003) (42) | 2 | 1.08 | 1.01 to 1.15 | 0.032 | 22.0%; 0.258 | FE |
| Complete improvement rate of pain in the limbs | Matthys (2003) (42) | 2 | 1.11 | 1.03 to 1.21 | 0.01 | 45.7%; 0.175 | FE |
| Complete improvement rate of dyspnea | Matthys (2007) (43) | 1 | 1.63 | 1.23 to 2.05 | < 0.001 | / | FE |
|  | Chuchalin (2005) (38) | 1 | 1.15 | 0.99 to 1.32 | 0.056 | / | FE |
| Number of patients unable to work on day 7 | Kamin(1) (2010) (39) | 4 | 0.43 | 0.25 to 0.76 | 0.004 | 95.0%; < 0.001 | RE |
|  | Kamin(2) (2010) (40) | 4 | 0.32 | 0.20 to 0.49 | < 0.001 | 80.5%; 0.002 | RE |
|  | Kamin (2012) (41) | 4 | 0.34 | 0.15 to 0.77 | 0.01 | 95.5%; < 0.001 | RE |
|  | Matthys (2003) (42) | 4 | 0.38 | 0.22 to 0.66 | 0.001 | 94.0%; < 0.001 | RE |
|  | Matthys (2010) (44) | 4 | 0.45 | 0.26 to 0.78 | 0.004 | 92.0%; < 0.001 | RE |
