# Supplementary material for: Flavonoids for Treating Viral Acute Respiratory Tract Infections: A Systematic Review and Meta-Analysis of 30 Randomized Controlled Trials
Source: Front Public Health. 2022 Feb 16;10:814669. doi: 10.3389/fpubh.2022.814669 (PMC8888526; doi:10.3389/fpubh.2022.814669)
Supplement: Supplementary file 2 [file Table_1.DOCX]

**Supplementary Appendix**

**PubMed**

**#1** Search “flavonoid” [MeSH Terms]

**#2** Search “respiratory tract infection” [MeSH Terms]

**#3** Search (flavonoid^*^ OR flavonol OR flavone OR flavanone OR flavan-3-ol OR anthocyanidin OR isoflavone OR quercetin OR kaempferol OR myricetin OR isorhamnetin OR luteolin OR apigenin OR hesperetin OR naringenin OR catechin OR epicatechin OR epigallocatechin OR epicatechin-3-gallate OR epigallocatechin-3-gallate OR cyanidin OR delphinidin OR malvidin OR pelargonidin OR petunidin OR peonidin OR genistein OR daidzein OR hespertin OR proanthocyanidin): Title/Abstract

**#4** Search (‘acute respiratory tract infection’ OR ARTI OR ‘respiratory tract infection’ OR virus OR respiratory OR URTI^*^ OR LRTI* OR RTI OR RTis OR respir$ OR ‘common cold’ OR ‘cold virus*’ OR colds OR influenza* OR flu OR ‘avian influenza’ OR ‘influenza A’ OR ‘influenza B’ OR ‘highly pathogenic avian influenza’ OR ‘seasonal influenza’ OR ‘Asian influenza’ OR ‘pandemic influenza’ OR ‘middle east respiratory syndrome coronavirus’ OR ‘severe acute respiratory syndrome’ OR ‘corona virus disease 2019’ OR H1N1 OR PH1N1 OR H3N2 OR AH1N1 OR H5N1 OR SARS OR MERS OR MERS-CoV OR COVID-19 OR coronavirus OR ‘corona virus’ OR SARS-CoV-2 OR 2019-nCoV OR n-CoV): Title/Abstract

**#5** Search (‘randomized controlled trial’ OR RCT OR randomized OR clinical OR trial): All Fields

**#6** Search **#1** OR **#3**

**#7** Search **#2** OR **#4**

**#8** Search **#6** AND **#7** AND **#5**

**Embase**

**#1** Search (‘acute respiratory tract infection’ OR ARTI OR ‘respiratory tract infection’ OR virus OR respiratory OR URTI* OR LRTI* OR RTI OR RTis OR respir$ OR ‘common cold’ OR ‘cold virus*’ OR colds OR influenza* OR flu OR ‘avian influenza’ OR ‘influenza A’ OR ‘influenza B’ OR ‘highly pathogenic avian influenza’ OR ‘seasonal influenza’ OR ‘Asian influenza’ OR ‘pandemic influenza’ OR ‘middle east respiratory syndrome coronavirus’ OR ‘severe acute respiratory syndrome’ OR ‘corona virus disease 2019’ OR H1N1 OR PH1N1 OR H3N2 OR AH1N1 OR H5N1 OR SARS OR MERS OR MERS-CoV OR COVID-19 OR coronavirus OR ‘corona virus’ OR SARS-CoV-2 OR 2019-nCoV OR n-CoV)

**#2** Search (flavonoid OR 'flavonol derivative' OR flavone OR flavanone OR 'flavan 3 ol' OR anthocyanidin OR isoflavone OR quercetin OR kaempferol OR myricetin OR isorhamnetin OR luteolin OR apigenin OR hesperetin OR naringenin OR catechin OR epicatechin OR epigallocatechin OR 'epicatechin 3 gallate' OR 'epigallocatechin 3 gallate' OR cyanidin OR delphinidin OR malvidin OR pelargonidin OR petunidin OR peonidin OR genistein OR daidzein OR hespertin OR proanthocyanidin):ti,ab,kw

**#3** Search ('randomized controlled trial' OR RCT OR randomized OR clinical OR trial)

**#4** Search **#1** AND **#2** AND **#3**

**Web of Science**

**#1** Search (‘acute respiratory tract infection’ OR ARTI OR ‘respiratory tract infection’ OR virus OR respiratory OR URTI* OR LRTI* OR RTI OR RTis OR respir$ OR ‘common cold’ OR ‘cold virus*’ OR colds OR influenza* OR flu OR ‘avian influenza’ OR ‘influenza A’ OR ‘influenza B’ OR ‘highly pathogenic avian influenza’ OR ‘seasonal influenza’ OR ‘Asian influenza’ OR ‘pandemic influenza’ OR ‘middle east respiratory syndrome coronavirus’ OR ‘severe acute respiratory syndrome’ OR ‘corona virus disease 2019’ OR H1N1 OR PH1N1 OR H3N2 OR AH1N1 OR H5N1 OR SARS OR MERS OR MERS-CoV OR COVID-19 OR coronavirus OR ‘corona virus’ OR SARS-CoV-2 OR 2019-nCoV OR n-CoV): Topic

**#2** Search (flavonoid OR flavonol OR flavone OR flavanone OR flavan-3-ol OR anthocyanidin OR isoflavone OR quercetin OR kaempferol OR myricetin OR isorhamnetin OR luteolin OR apigenin OR hesperetin OR naringenin OR catechin OR epicatechin OR epigallocatechin OR epicatechin-3-gallate OR epigallocatechin-3-gallate OR cyanidin OR delphinidin OR malvidin OR pelargonidin OR petunidin OR peonidin OR genistein OR daidzein OR hesperetin OR proanthocyanidin): Topic

**#3** Search (clinical OR trial OR randomised OR randomized OR random): Topic

**#4** Search (mice OR mouse OR rat OR rats): abstract

**#5** Search **#1** AND **#2** AND **#3** NOT **#4**

**Cochrane Library**

**#1** Search MeSH descriptor: [Flavonoids] explode all trees

**#2** Search (flavonoid OR flavonol OR flavone OR flavanone OR anthocyanidin OR isoflavone OR quercetin OR kaempferol OR myricetin OR isorhamnetin OR luteolin OR apigenin OR hesperetin OR naringenin OR catechin OR epicatechin OR epigallocatechin OR cyanidin OR delphinidin OR malvidin OR pelargonidin OR petunidin OR peonidin OR genistein OR daidzein OR hespertin OR proanthocyanidin):ti,ab,kw

**#3** Search MeSH descriptor: [Respiratory Tract Infections] explode all trees

**#4** Search (“acute respiratory tract infection” OR ARTI OR “respiratory tract infection” OR virus OR respiratory OR URTI* OR LRTI* OR RTI OR RTis OR “common cold” OR “cold virus*” OR colds OR influenza* OR flu OR “avian influenza” OR “influenza A” OR “influenza B” OR “highly pathogenic avian influenza” OR “seasonal influenza” OR “Asian influenza” OR “pandemic influenza” OR “middle east respiratory syndrome coronavirus” OR “severe acute respiratory syndrome” OR “corona virus disease 2019” OR H1N1 OR PH1N1 OR H3N2 OR AH1N1 OR H5N1 OR SARS OR MERS OR MERS-CoV OR COVID-19 OR coronavirus OR “corona virus” OR SARS-CoV-2 OR 2019nCoV OR n-CoV):ti,ab,kw

**#5** Search **#1** OR **#2**

**#6** Search **#3** OR **#4**

**#7** Search **#5** AND **#6**

**ClinicalTrials.gov registry**

**#1** Search (‘acute respiratory tract infection’ OR ARTI OR ‘respiratory tract infection’ OR virus OR respiratory OR URTI OR LRTI OR RTI OR RTis OR ‘common cold’ OR ‘cold virus’ OR colds OR influenza OR flu OR ‘avian influenza’ OR ‘influenza A’ OR ‘influenza B’ OR ‘highly pathogenic avian influenza’ OR ‘seasonal influenza’ OR ‘Asian influenza’ OR ‘pandemic influenza’ OR ‘middle east respiratory syndrome coronavirus’ OR ‘severe acute respiratory syndrome’ OR ‘corona virus disease 2019’ OR H1N1 OR PH1N1 OR H3N2 OR AH1N1 OR H5N1 OR SARS OR MERS OR MERS-CoV OR COVID-19 OR coronavirus OR ‘corona virus’ OR SARS-CoV-2 OR 2019-nCoV OR n-CoV)

**#2** Search (flavonoid OR flavonol OR flavone OR flavanone OR anthocyanidin OR isoflavone OR quercetin OR kaempferol OR myricetin OR isorhamnetin OR luteolin OR apigenin OR hesperetin OR naringenin OR catechin OR epicatechin OR epigallocatechin OR cyanidin OR delphinidin OR malvidin OR pelargonidin OR petunidin OR peonidin OR genistein OR daidzein OR hespertin OR proanthocyanidin)

**#3** Search **#1** AND **#2**
